# Exosomes and ultrasound: The future of theranostic applications

**DOI:** 10.1016/j.mtbio.2023.100556

**Published:** 2023-01-20

**Authors:** Badrinathan Sridharan, Hae Gyun Lim

**Affiliations:** Department of Biomedical Engineering, Pukyong National University, Busan, 48513, Republic of Korea

**Keywords:** Exosomes, Extracellular vesicles, Ultrasound, Acoustics, Theranostics

## Abstract

Biomaterials and pertaining formulations have been very successful in various diagnostic and therapeutic applications because of its ability to overcome pharmacological limitations. Some of them have gained significant focus in the recent decade for their theranostic properties. Exosomes can be grouped as biomaterials, since they consist of various biological micro/macromolecules and possess all the properties of a stable biomaterial with size in nano range. Significant research has gone into isolation and exploitation of exosomes as potential theranostic agent. However, the limitations in terms of yield, efficacy, and target specificity are continuously being addressed. On the other hand, several nano/microformulations are responsive to physical or chemical alterations and were successfully stimulated by tweaking the physical characteristics of the surrounding environment they are in. Some of them are termed as photodynamic, sonodynamic or thermodynamic therapeutic systems. In this regard, ultrasound and acoustic systems were extensively studied for its ability towards altering the properties of the systems to which they were applied on. In this review, we have detailed about the diagnostic and therapeutic applications of exosomes and ultrasound separately, consisting of their conventional applications, drawbacks, and developments for addressing the challenges. The information were categorized into various sections that provide complete overview of the isolation strategies and theranostic applications of exosomes in various diseases. Then the ultrasound-based disease diagnosis and therapy were elaborated, with special interest towards the use of ultrasound in enhancing the efficacy of nanomedicines and nanodrug delivery systems, Finally, we discussed about the ability of ultrasound in enhancing the diagnostic and therapeutic properties of exosomes, which could be the future of theranostics.

## Graphical abstract

**Figure:**
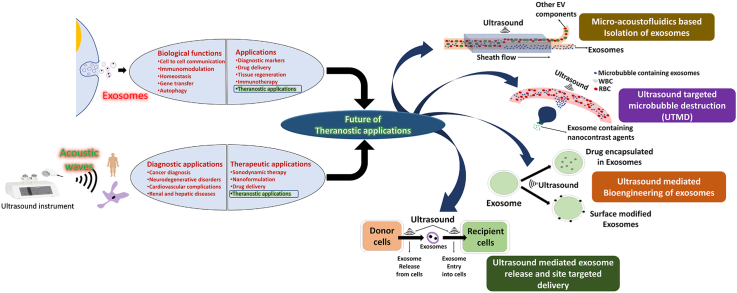

## 1 Introduction

Human system consist of several cell types and all the cells are needed to be in synchrony for normal physiological function. Hence, a constant mode of cell-cell communication is maintained between functionally related cells and extracellular vehicles (EVs) are one among the cellular cargo systems that play a very prominent role in intercellular communications [1,2]. It was believed that EVs provide key information about the cellular health as they are secreted by both normal cells and cells under pathological stress [3,4]. Microvesicles, apoptotic bodies and exosomes are different types of EVs classified based on size and mode of secretion [5,6]. Exosomes are bi-layered vesicles, which range between 30 and 150 ​nm and are released by well-regulated exocytosis process. They were initially considered as cellular waste disposal system, but recent studies provided comprehensive evidences that exosomes carry diverse range of biomolecules that can regulate the cellular metabolic process which are reflected as altered physiological or pathological processes. These effector molecules from exosomes can be potential biomarkers for specific diseases [7].

Exosomes were first identified as small vesicles ranging around 100 ​nm, secreted by chondrocytes and later it was identified that they are part of EVs secreted by platelets [8,9]. Further exosomes were identified in subsequent years from osteogenic cells that help in bone and tooth formation [10,11]. It was believed that, similarly functioning exosomes were involved in both normal physiological functions like wound repair, bone formation etc. [[12], [13], [14]], and pathological processes of thrombus induced disorders and certain autoimmune diseases [[15], [16], [17]]. Research studies in early 1980s have showed exosome secretion during differentiation of reticulocytes and also in seminal fluids that help in sperm maturation [[18], [19], [20]]. Collectively the early studies in identification of exosome and its mechanism of secretion revealed that they are considered as protein quality control and thus exosomes contain significant information about the health status of the cells and allows them to contribute to health and disease management [21].

Exosomes are similar to a cell in the structural characteristics and the size of exosomes released, mainly depend on their composition and health status of the cells [22]. On the other hand, biochemical heterogeneity of exosomes is very common, as they contain almost all types of biomolecules such as proteins, enzymes, glycans & glycoconjugates, lipids derivatives and nucleic acids [7]. After several studies done with exosomes, certain proteins that commonly occur in exosomes of different cellular origin, are considered as specific markers of exosomes. Certain physiological and pathological functions of exosomes are carried out by lipid and nucleic acid contained in it [23].

**Fig. 1:**
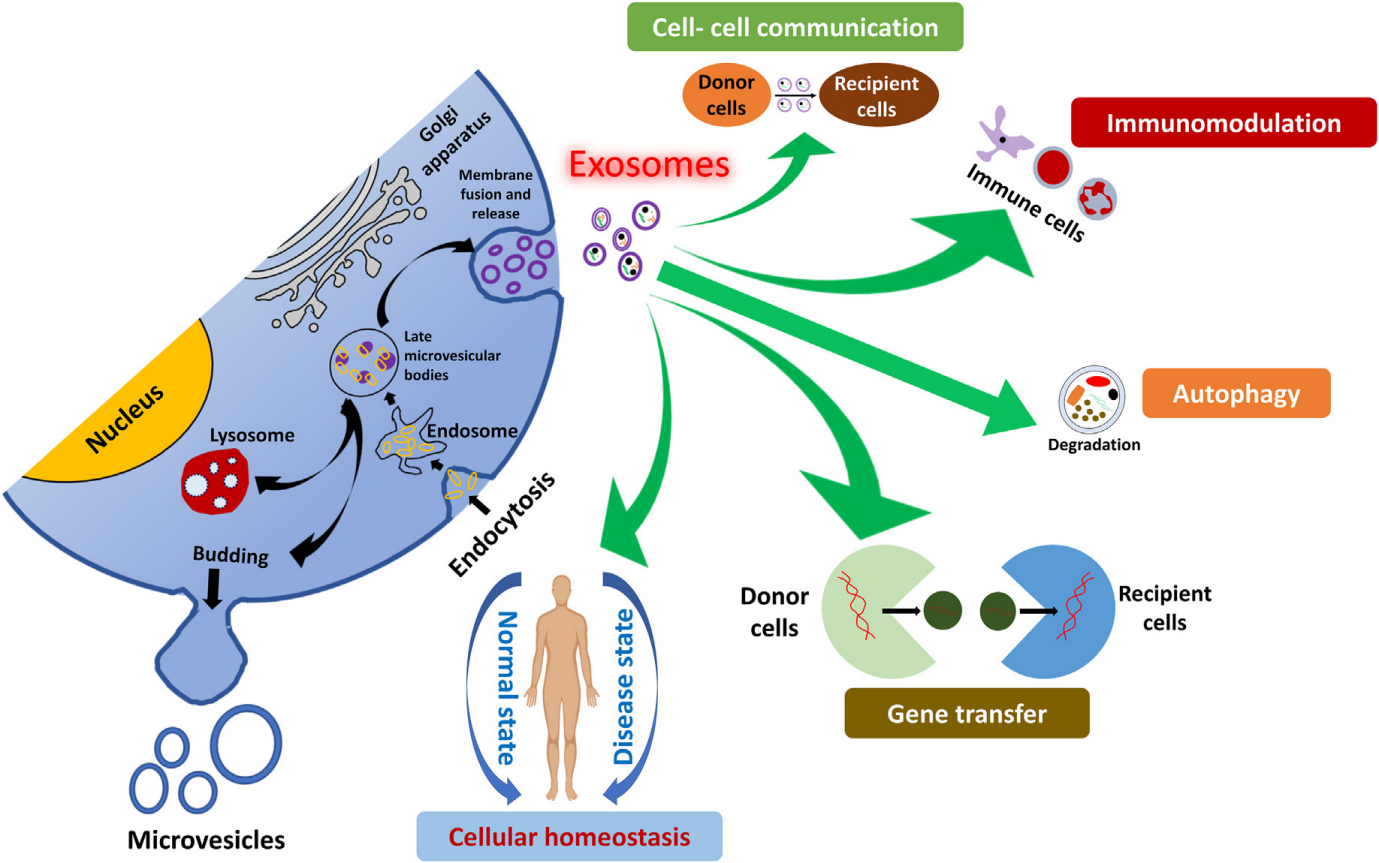
Biological functions of exosomes [35].

Several functions of exosomes were reported continuously since its discovery and among which, strong evidences for cellular waste management and intercellular communications by exosomes were established (Fig. 1). Exosomes are believed to be the quality control of protein in the cells. Plasma membrane budding of zygotes induced by fertilization to remove the sperm receptor is one of the best examples of protein waste removal [[24], [25], [26]]. Further it was believed that exosomes also carry out protein sorting pathway that help in defining the anterior-posterior polarity of migrating cells that includes from simple amoeba to highly defined human leucocytes [27,28]. Another important function of exosomes is the cell-to-cell communication, in which they carry signaling molecules that deliver information from distant cells by either cell uptake or by activating the receptors in the recipient cells’ surface, that can regulate the desired metabolic and signaling pathway [29]. Exosomes also help in remodeling of extracellular matrix that result in physiological or pathological processes. Wound repair and osteogenesis are common physiological processes that are significantly derived by exosomes and its influence in ECM remodeling [[30], [31], [32]]. On the other hand, cancer cell plasticity, pathological calcification and neurodegeneration were also mediated by exosomes [33,34].

The biological functions of exosomes make it highly desired medium for understanding the physiological status of the cells and progression of the pathological conditions. Exosomes has gained so much of attention among the multidisciplinary researchers recently as they are instrumental in biomarker/image guided diagnosis of specific diseases [36,37]. The ability of the exosome-derived molecules that either get internalized in the recipient cells or regulate cellular pathways through receptors make them ideal candidate for an efficient drug delivery system [38]. These properties of exosomes were considered by the biomedical researchers in developing exosomes as a potential theranostic agent. There are several challenges involved in materializing the applications of theranostic agents including the key factors like specificity, site targeting, etc. This led the experts to seek for an external triggering force or image guidance to potentiate the theranostic properties of exosomes and other similar theranostic agents. Imaging modalities such as MRI, CT, ultrasound etc., were commonly sorted for image guided diagnostic and therapeutic applications [37].

Biomedical imaging has created a specific niche in disease diagnosis and had improvised several therapeutic strategies for certain complex conditions such as cancer and neurodegeneration [39,40]. Certain physical properties of biological molecules such as magnetism, acoustics, ionization, radiation, fluorescence etc., were utilized to provide significant information about the pathological and anatomical status of the system for diagnosis of the disease condition [41]. Though every imaging method has its own advantages, ultrasound was one of the commonly prescribed non-invasive imaging methods, because it does not require high doses of contrast agent like MRI for better visualization, does not involve ionizing radiations, but provide significant information about the soft tissues [35,42].

Different cells respond to ultrasound uniquely and are effective in modulating the migration and mobility of the cells [43]. Apart from the physical properties, intra/extracellular molecular profile of cells changes due to acoustic manipulations. Several researchers focus and explore on exosomes as one of the media that are being used for improving the cellular uptake of the drug and diagnosis or tracking of specific cell types. The influence of ultrasound over cellular properties was exploited by several researchers to focus on modifying exosomal secretion or exosome mediated drug delivery [44]. In this review, we would like to discuss about the biological role of exosomes and how they are utilized to improve the drug delivery and diagnosis. Further, the ability of ultrasound in tweaking the cellular characteristics will be explored in detail and provide evidence and perspectives in support of how ultrasound will improve the theranostic property of exosomes.

## 2 Biochemistry of exosomes

Exosomes consists of a collection of molecular mediators that acts as a medium of communication between several cell types in the biological systems (Table 1). They require certain biomolecules for their structural integrity and to carry out their biological function. Since, they are released apart from the cells, they possess molecules that are byproducts of the metabolism or signaling pathways including the metabolites that are involved in them [21]. According to ExoCarta (http://www.exocarta.org), a database of exosomal biochemical profile, there are around 10,000 proteins, 1100 lipids and more than 6000 RNA derivatives were identified among which 3400 mRNAs and 2800 miRNAs [5,45,46].

**Table 1:** Biochemical profile of exosomes.

Type of biomolecule	Name of the biomolecule	Host cell function	Exosomal function	Reference
Protein	Tetraspanins (CD81, CD82, CD37, CD63)	Organization of the membrane and intracellular protein in to microdomain of intercellular adhesion, signaling and adaptor and	Trafficking of other proteins (MHC class II, ICAM-1, SDC1-4, Ig family) through exosomes Inclusion of integrins by tetraspanins, play important role in precancerous lesion development	[47,48]
	Viral membrane proteins (Envelop proteins)	Viral replication in the infected host cells	Virus utilizes exosome biogenesis for viral assembly and transmission Influence exosomal function in immunosuppression	[49,50]
	Immunosuppressive proteins (PD-L1 & CD 100)	Immunosuppression by interacting with immune cells	Reprogramming the immune system far from tumor site	[51,52]
	Integral membrane signaling proteins/growth factor receptors (EGFR, SDFR, VEGFR, Cytokine receptors, T cell receptors, GCPR, Notch receptors)	Phosphorylation of tyrosine residues from various intracellular signaling molecules	Surface signaling in the recipient cells and delivery of functional receptors to cells where the specific proteins were downregulated	[53,54]
	Lipid-anchored proteins (GPI-anchored proteins, ectonucleotidases, complement inhibiting proteins, cellular prion proteins, glypican-1, prenylated, myristoylated, palmitoylated proteins)	Adhesion, hydrolysis, complement regulation, embryogenesis, apoptosis, neurotransmission, cellular growth and differentiation	Key roles in cancer progression	[[55], [56], [57], [58]]
	Enzymes (CD39, CD73, phosphatases, pyrophosphatases, annexins, phosphate transporters, RNA editing enzymes)	Catalysis of physiologically important reaction at intracellular and systemic level	Energy metabolism	
	Peripheral Surface Proteins (Wnt proteins, bone morphogenic proetiens, TGF-β, TNF-α. FAS ligand, TRAIL, extracellular matrix proteins-Fibronectin, tenascin C, etc.)	Cell growth and development, apoptosis, Bone formation, morphogenic signals, immunomodulation, extracellular matrix formation and cell integrity	Surface signaling and delivery to the recipient cells	
Lipids and derivatives	Phospholipids	Intracellular signaling, cellular integrity and protection	Exosomal membrane structure	[[59], [60], [61], [62]]
	Ceramides	Induction of apoptosis, skin hydrophobicity and protection, hormonal function in insulin related pathway		
	Glycosphingolipids/gangliosides	Neurological function and cell membrane of the CNS		
	Cholesterol	Membrane structure, intracellular signaling, precursors for steroid hormones		
Carbohydrates and derivatives	Sialic acids	Intercellular interactions, carbohydrate-protein interaction, tumor metastasis, bacterial/viral inhibition	Tumor progression	[63]
	Hyaluron sulphate	Wound healing, carbohydrate-protein interaction, tumor progression		[64,65]
	Heparan sulphate	Wound healing, host defense, energy metabolism, morphogenesis		[66]
Nucleic acids	RNA (Oct-4 mRNA, ncRNA, snRNA, tRNA, miRNA)	Regulation gene expression for cellular signaling	RNA quality control, Cellular signaling in progression of glioblastoma cells and mast cells	[[67], [68], [69]]
	DNA (dsDNA, ssDNA, mitochondrial DNA)	Gene expression	DNA quality control, chemoresistance of cancer cells, viral infection	[70,71]

The biochemical content of exosomes significantly affects the physical properties like shape, size, and density of exosomes [37]. Density of an exosome is greatly affected by the protein to lipid ratio and increased expression of a single cargo protein in exosome greatly increases the density of the exosomes. This in turn greatly influences the size and shape of the exosomes and can provide valid evidence for exosomes occurring in varied sizes even from the same cell [72,73]. Each cell type in a biological system release exosomes with different size and shapes, which is a result of the biochemical content expressed in the exosomes. Though certain biomolecules expressed in the exosomes are commonly identified, some of them are highly conserved for each cell type. This has paved way for clinicians to focus on the biochemistry of exosomes for identifying biomarkers for specific diseases. Several specific biomarkers were identified from exosomes of varied cell type that helps greatly in diagnosis of respective diseases. Similarly, the size and shape influenced by the biochemical content also play a vital role in theranostic properties of exosomes. The physico-chemical property of exosomes was also managed by the surface molecules like the lipid bilayer, proteins, and sugar residues of the glycoproteins. However, considering the therapeutic potential of exosomes, the drawbacks like heterogenicity in size and poor systemic stability are countered by engineering the surface of the exosomes with several molecules which might help in elevating the theranostic potential of exosomes [[74], [75], [76]]. In the following section, the efficacy of exosomes with its inherent/acquired ability, to render its theranostic property and how the challenges faced by current theranostic agents were addressed through exosomes.

## 3 Theranostic applications of exosomes

Exosomes being responsible for intracellular communication and biomolecular cargo system, are believed to add significant value to the diagnostic and therapeutic applications by increasing the specificity and efficacy [23]. Exosomes are known to be utilized as such in identification and prognosis of certain complex metabolic diseases like cancer, neurodegeneration, fibrosis etc., that are originated from different organ or cells [64]. Generally, the exosomes from liquid biopsies are more helpful in diagnostic applications as the sampling techniques are relatively easier and minimally invasive. This helps experts to rely on exosomes-based diagnosis for a longer duration and produce a complete prognostic report of patients [65]. On the other hand, therapeutic property of exosomes can be achieved either by intact exosomes or exosomes loaded with certain desired molecules (macro/micro molecules) to exert therapeutic action on desired target cells [66,67]. There are certain reports that supports the use of exosomes as theranostic agent which will be discussed in this section [37]. Significant exploration has gone into exploiting the target cell specificity of exosomes for efficient drug delivery and increasing the kinetics of certain active pharmaceutical ingredients [68].

Research and development in understanding the biochemistry of exosome (biogenesis, release and signaling pathways influenced in the recipient cells) either in naïve form or engineered form, has provided opportunities for a wide range of applications that includes image-guided site targeting and enhanced drug delivery [36,69]. Better understanding of biophysical and biochemical characteristics has helped exosomes to venture into theranostic platforms. Exosomes are known for presence of important biomarkers, that can provide information about the stage and severity of the disease. Similarly, using specific engineered exosomes as contrast agents, where damaged/disease prone cells/tissues can be selectively observed and targeted among the healthy tissues. Fundamental requirement for approaching exosomes as a theranostic agent is to achieve loading the therapeutic/diagnostic agent in the core or surface of the exosomes and to avoid systemic distribution of the disease-causing exosomes [23,66]. The following section provided information about the above-mentioned applications of exosomes in detail with reported studies and their prospective applicability. Diverse application of exosomes in biology and medicine was summarized in Table 2.

**Table 2:** Clinical and pharmaceutical applications of exosomes.

Type of application	Specific biomolecules involved	Specific disease condition	Reference
Diagnosis	CD81	Hepatitis mediated liver fibrosis	[77]
	CD63/LAMP-3	Melanoma, lung and ovarian cancer	[78,79]
	EGFR	Glioblastoma	[57,80,81,82,83,84,85]
	Glypican-1, miR-375 miRNA-200c-3p, 21–5p and Let-7a-5p	Pancreatic cancer Pancreatic cancer severity scoring	
	Retinoic acid induced protein/Resistin	Bladder cancer	
	miRNA-21	Esophageal lesions	
	mi-RNA-139–5p, 378a, 379 and 200–5p	Lung cancer	
	miRNA-574–3p and 141–5p	Prostate cancer	
	piR-4987	Lymph node metastasis	[[86], [87], [88]]
	piR-932 and PIWIL2	Breast cancer metastasis	
	piR-32052, 39,894 and 43,607	Renal carcinoma	
	Aβ-peptide and phosphorylated τ-protein	Alzheimer's disease	[89,90]
	cathepsin-D and α-synuclein	Parkinson's disease	[90,91]
Therapy	KrasG12D-specific siRNA	Lung carcinoma	[92]
	Mesenchymal stem cells derived exosomes	Host Vs Graft rejection	[93]
	Umbilical cord-mesenchymal stem cells derived exosomes	Immunomodulation for treatment of diabetes	[94]
	Exosomes from T-regulatory cells		[95]
	Neurological cell derived exosomes	Brain ischemia, Parkinson's disease	[96,97]
	Viral infected cells derived exosomes	SARS-CoV-2	[98,99]
Site targeted therapy	Viral infected cells derived exosomes loaded with target protein through HIV-Nef, Env protein, Nedd4	HIV infection (AIDS)	[100]
	Transferrin & lactoferrin	Pre-cancerous lesions	[101]
	Ubiquitination	Therapy based on target proteins	[[102], [103], [104]]
	Myristoylation and palmitoylation		
	L7Ae and C/D box		[105,106]
	Connexin 43		[107]
	miR-193a bound to Major vault protein, myoferlin, AGO2 and GW182	Tumor suppression	[108]
	miR-126	Myocardial ischemic injury	[109]
	anti-miR-214 gene	Gastric tumor sensitization and inhibition of metastasis	[110]
	HGF-siRNA	Tumor metastasis	
Drug delivery	Mesenchymal cells derived miR-124	Glioblastoma	[111]
	Prostate cancer cells derived miR-141–3p	Bone metastasis	[112]
	Integrin αVβ5	Liver lesions	[113]
	Integrin α6β1	Lung cancer	
	Lactadherin C1C2 domain and Lamp2b	Tumor inhibition	[114]
	Immature dendritic cell derived exosomes with iRDG and Lamp2b	Breast cancer inhibition	[115]
	rabies virus glycoprotein (RGV) and related peptides; c(RGDyK)	Brain lesions	[116]
	Morphine receptor silencing siRNA bound to RGV	Morphine addiction	[117]
	Klotho gene and adenosine kinase siRNA	Endothelial dysfunction	[118]
	Glycosylphosphatidyl inositol (GPI)	Immunomodulation	[119]
	A33 antibodies with iron oxide nanoparticles	Inhibition of tumor growth	[120]
	Aminoethyl-anisamide-polyethylene glycol paclitaxel	Cancer cell accumulation and inhibition of tumor growth	[121]
	Antibodies-like RNA	Prostate cancer, breast cancer or colorectal cancer cells	[122]
	Small molecule inhibitors or siRNA with antibodies-like RNA	Cancer inhibition	
	Glycoprotein based adhesion molecules	Wound repair	[123]
	PEG with phospholipid derivative	Targeting epidermal growth factor receptors	[124]

### 3.1 Biomarkers identified from exosomes

Cells involved in pathogenesis secrete exosomes and exosomal biomolecules are thus utilized as diagnostic and prognostic markers. Similarly, exosomes from normal cells also provide their health status [70,71]. Exosomes were released during several pathogenic process such as inflammation, immune response, cancer progression, cell damage and death [125]. The released exosomes from the cells are either obtained by culturing ex vivo or it can be isolated from the body fluids like blood, urine, saliva, sweat etc. The possible biomolecules identified in exosomes like transmembrane/intracellular proteins and nucleic acids are considered as biomarkers that can provide useful information for diagnosis, staging of the disease and prognostic process before/after therapy [23,126]. Despite tremendous success in exosomal biomarker-based diagnosis of different diseases, notable drawback is also identified such as loss of molecular integrity in the exosomes due to improper storage conditions. Storing the exosome containing samples below −70 ​°C was considered optimum and storage conditions for exosomes are still being optimized. The basic biochemistry and specific biomarkers for disease diagnosis and staging is still being explored and hence, a single biomarker for appropriate disease staging is still unidentified [127].

Tetraspanins are the well-established biomarkers detected in exosomes from variety of cell types or body fluids. Increased CD81 was correlated with viral hepatitis induced fibrosis in liver [128]. Cancer diagnosis is one of the best examples for exosomes as a diagnostic marker, as several proteins were identified and established for their occurrence in exosomes [33]. These identified proteins are well-known for its cellular signaling such as cell adhesion, migration, and trafficking [129]. Another significant information obtained from various studies are that during cancer progression, the number of exosomes produced by the cancerous cells is more than the normal cells [130]. Tumor growth influenced by certain important pathogenic steps like angiogenesis, and metastasis, are reported to be triggered by tumor derived exosomes. CD63 also known as lysosomal associated membrane protein 3 (LAMP-3) was reported to be upregulated during melanoma, lung, and ovarian cancer conditions [131,132]. Similarly, elevated exosomal occurrence of epidermal growth factor receptor (EGFR) during glioblastoma, glypican-1 at an early stage of pancreatic cancer and retinoic acid-induced protein 3, resistin etc., during bladder cancer are some of the best examples of exosome derived markers for the specific type of cancers [49,133,134]. Similar to exosome-derived proteins, nucleic acids in exosomes, especially micro-RNA (miRNA), small interfering RNA (siRNA), p-element-induced wimpy testis (PIWI)-interacting RNA (piRNA), contribute to the diagnosis of cancer. Analysis of circulating miRNA during cancer diagnosis apart from tissue biopsy, is considered very effective in detection of type and stage of cancer [[77], [78], [135], [136]]. Some of the reported mi-RNAs include miRNA-21 for esophageal lesions, miRNA-139–5p, 378a, 379 and 200–5p for lung cancer, miRNA-574–3p and 141–5p for prostate cancer [79,80]. It is not just mere diagnosis that mi-RNA aid in cancer management, but they were very effective in differentiating the lesions and identify the specific type of cancer [[81], [137], [138]]. miRNA-375 isolated and analyzed from plasma derived exosomes were able to differentiate pancreatic cancer from benign hyperplasia. It was also reported to help in identifying the progression of inflammation to oral carcinoma and can be a potential early-stage biomarker. miRNA-200c-3p and 21–5p were analyzed to differentiate the stage of pancreatic cancer and provide severity score, along with the help of Let-7a-5p miRNA [139,140]. Apart from this, exosomes obtained from human amniotic fluid, saliva and urine consists of several miRNA molecules and they provide information about chance of prenatal renal failure and pancreatic cancer [82,83]. Identification of bare mRNA in urinary exosomes with upregulated glycoprotein enzymes, a prostate specific antigen provided diagnosis of prostate cancer and reported to reduce the biopsy sampling of almost 25% of the patients [141]. piRNA derived from exosomes is another efficient tool for diagnosis of various disease which has pathogenic process at the genetic level as piRNA plays crucial role in gene silencing, germ stem cell maintenance and they are oncogenic. Somatic cells secrete exosomes with less piRNA concentration and when these cells undergo cancerous modifications, it influences the level of piRNA in the exosomes secreted by them and they aid in different stages of cancer progression from proliferation, inhibition of apoptosis to metastasis, and invasion [[84], [142], [143]]. piRNA-4987 provides diagnosis of lymph node metastasis, while piRNA-932 and PIWIL2 indicate breast cancer metastasis. During renal cell carcinoma increased levels of piRNA-32052, 39,894 and 43,607 in exosomes were observed and these molecules provide information about the clinical stage of the cancer progression and survival rate of the patients [[85], [144], [145]]. Apart from cancer, certain neurodegenerative diseases were also diagnosed using the exosomal markers such as increase Aβ-peptide and phosphorylated τ-protein in neural cell derived exosomes indicate the Alzheimer's disease [146,147]. Similarly, increased cathepsin-D and α-synuclein levels are proposed to indicate the early stage of Parkinson's disease progression [147,148]. Research studies are being conducted to upgrade and develop the exosome-based diagnosis for cancer and other complex diseases with respect to specificity. Deeper exploration to understand the intracellular sorting of exosomal molecules, extracellular release and internalization in the donor cells can provide valid insights about the pertaining disease conditions and can make exosomes potential to analyze the early onset and staging of the diseases [7,149,86].

### 3.2 Engineered and naive exosomes for therapeutic applications

Predominant applications of exosomes were reported to be in the diagnostic platform until many of those reports were translated to clinical set up. Despite being potential to influence the physiological and molecular signaling exosomes, there are noticeable number of research reported on their therapeutic applications, which are still at preclinical stage and only very few of the studies are being tried in clinical trials. However, several interesting studies were reported in the past decade with respect to therapeutic potential of exosomes either in naïve or engineered form for treatment of certain disease conditions like cancer, immune related disorders, neurological disorders, and infection [67].

Exosomes from tumor cells in their naïve form are loaded with certain influencing biomolecules and are used in cancer therapy. Exosomes from patient's own tumor cells in naïve form were used to enhance the innate immunity and avoid immune evasion by cancer cells [23]. It was presumed that insulin like growth factor receptor when loaded on to glioma cells obtained from same patient, it might inhibit tumor progression by interacting with tyrosine kinase receptors. This study was a clinical trial and the results were not disclosed [87]. Similarly, several trials involving exosomes from mesenchymal stem cells were utilized to treat pancreatic cancer by loading it with KrasG12D-specific siRNA, which targets KRAS oncogene [88]. Tumor antigen loaded dendritic cell exosomes were test clinically against lung cell carcinoma. Some of the studies did not show very effective response with respect to tumor inhibition [23,89,90]. Exosomes showed their potential in immunomodulation and anti-inflammation, which are key signaling pathways in many disease conditions influenced by immune cells [91]. MSC derived exosomes were effective against graft Vs Host disease through inhibition of proinflammatory reactions and cytokine responses [150]. Clinical trials for diabetes and macular degenerations have adopted this hypothesis. Similarly, umbilical cord MSCs were isolated to obtain their exosomes for treatment of diabetes through immunomodulation in β-cells [151]. T-regulatory (T-reg) cells has the ability to restore the T-helper cells based immune balance and this phenomenon was used to treat diabetes type-1 by modulation of T-reg cells activity effected by cord-blood stem cells derived exosomes [152]. In case of neurological disorders, exosomes possess a significant advantage of the ability to cross blood-brain barrier. Neurodegenerative conditions like ischemic stroke and Parkinson's diseases were effectively treated with exosomes, specifically with the ones that has ability to modulate inflammatory process [[92], [153], [154]]. Several deadly infections including current pandemic caused by SARS-CoV-2 were also reduced by exosomes and exosomes-based vaccines [155]. Exosomes isolated from infected cells were used to deliver disease-associated antigens. This approach was adopted to study SARS-CoV-2 inhibition *in vitro* and clinical trials were initiated against pulmonary injuries caused by COVID-19 infections [93,94]. Dendritic cell-exosomes were loaded with *Toxoplasma gondii* antigen to tweak the immune response and effectively treat pertaining infections [95].

The inconsistent results obtained with the naïve exosomes showed that, major modification in the exosomes by loading with protein, nucleic acids, and small molecules, can impact some important intracellular events involved in disease progression at molecular level [96]. Proteins were incorporated on to the exosomes by inducing the over expression of specific protein in donor cells for inclusion in exosomes, or including the target proteins along with the biomolecular milieu of exosomes, modifying the target protein and external force assisted protein inclusion [[97], [156], [157]]. Target proteins are over expressed by including the specific gene producing target protein was transfected into the donor cells and isolated in the secreted exosomes. Though this strategy is straight forward and the engineering process is carried out naturally, there are several issues that are to be addressed like cytotoxicity due to abnormal target protein concentration, low specificity, and chance of inappreciable biological response [157]. In order to overcome these limitations constant explorations are ongoing to incorporate the target protein specifically for over expression and recipient cell uptake. One of the strategies to specifically incorporate the target protein is by fusion along with protein milieu in the exosomes. Fusion of mutated target protein with HIV-1 Nef protein helped in assembly of the target protein in the exosomes [98]. There are certain studies which involved in aiding the target protein by the exosome sorting machinery (ESCRT). Nedd4 family-interacting protein 1 and cytosolic domain of Env protein are some of the protein subunits well recognized for exosomal packaging and makes it suitable target for fusion of target proteins [99]. Transferrin and lactoferrin uptake by precancerous tissues was successfully delivered by fusing with GAPDH and inclusion in the exosomes [158]. Currently several researchers focus on successfully fusing the target protein with the exosomal proteins, without altering the biological function of the target protein. Mostly fusion of target proteins with peptides, rather than complete proteins were considered for exosomal packing. Apart from fusion of target proteins, modification in the expressed target proteins was also another successful therapeutic strategy [159]. Ubiquitination of the target protein has shown significant increase in the exosomal target protein concentration. Mono-ubiquitinated syntaxin 3, sorting of MHC class II β chain are examples of ubiquitin mediated exosomal sorting of target proteins. Myristoylation and palmitoylation were also found to enable the packaging of target protein in to exosomes, similar to ubiquitination [[160], [161], [162]]. Certain externally applied mechanical force also aided in inclusion of target proteins in to exosomes, where the mechanical force includes repeated freeze thaw cycles, sonication, saponin based permeabilization and mechanical extrusion which were successful in loading catalase in to exosomes for Parkinson's disease management [100]. The protease degradation of the target proteins was achieved by sonication, mechanical extrusion and permeabilization more efficiently with increased protein loading and sustained release from exosomes. Major concerns with this method of protein loading are the difficulty in exosome purification after engineering and maintenance of exosomal integrity [157,163].

Nucleic acids that can affect the disease progression at the molecular level, was also explored for its inclusion in to the exosomes. Exogenous siRNA inclusion with exosomes was tried *in vitro* with HeLa cells and showed a good loading efficiency. This method includes direct incubation of exosomes with the commercially procured siRNA molecules and found to be fairly straight forward [101]. However, several RNA molecules which pose challenge during the loading process were achieved by electroporation, molecular guidance through protein and peptides that has ability to bind to specific RNA sequences and can be achieved by certain helper molecules for cytosolic delivery that are conserved for exosomal packaging [157]. L7Ae, an archaeal ribosomal protein was bound to CD63 at the *C*-terminal region and adding C/D box into the gene of interest at 3′-UTR in the RNA structure. The gene of interest was internalized in the exosomes through the L7Ae and C/D box [164,102]. Similarly, Connexin 43 (Cx43) was packaged in to the exosomes and mutation of S368A of the protein sequence greatly help in delivery of Cx43 in to the target cells. This mutated Cx43 can be exploited for cytosolic translocation of desired protein through exosomes. C/D box and related gene manipulation are together called RNA packaging device and with the help of cytoplasmic transfer agents, genes were transfected to the donor cells to produce the engineered exosomes loaded with desired proteins [103]. Exosomal-RNA analyses revealed that three motifs are considered as signature sequences that are conserved for most of the exosomes. These sequences were targeted to generate candidate RNA to get internalized into exosomes [104]. Tumor derived exosomes were detected with high levels of tumor suppressive miRNA (miRNA-193a) and they were noted to interact with a protein called major vault protein (MVP) without which the desired miRNA involved in inhibition of cancer progression, was found to be accumulated in the intracellular compartment [165]. Myoferlin, AGO2 and GW182 are some of the proteins identified with similar property like MVP and they are continuously explored *in vitro* and *in silico* for nucleic acid packaging into the exosomes [166]. Other approaches include transfection of desired miRNA or siRNA into the cells and natural accumulation in exosomes. Therapeutic role of miRNA-126 against ischemic injury in myocardium, miRNA-214 inhibition carried out by exosomal anti-214 leading to gastric tumor sensitization for chemotherapy and inhibition of tumor metastasis by exosomal delivery of HGF siRNA are some of the best examples of transfection of desired nucleic acids into the donor cells through exosomes [167,105]. Loading large sequence of RNA into the exosomes is quite difficult and hence, relying on the gene editing system (CRISPR/Cas9) in the recipient cells is not a feasible approach. However, preparation of hybrid containing liposome encapsulated exosomes are developed which showed great promise in enhanced large RNA/DNA accumulation and delivery into the recipient cells [106].

There are certain drug molecules loaded directly in to the exosomes using methodologies like mixing and incubation, ultrasonic treatments and etc., [107]. Some of them includes, curcumin incubation with the exosomes, loading chemotherapeutic drugs like paclitaxel into exosomes using electroporation, ultrasonic waves and through direct mixing [168]. Stability of the drug, its site-specific release and efficacy were tested with these three exosome engineering methodologies, where ultrasonic treatment showed the best results [157,108]. Direct incubation of paclitaxel with the donor cells was performed and it resulted in secretion of exosomes accumulated with this drug [169]. Freeze-thaw cycles, membrane permeabilization using saponins were also effective in internalization of drug molecules into the exosomes for Parkinson's disease treatment [100]. Major disadvantage of this strategy of exosome engineering is difficulty in isolation, concerns with stability of drug inside the exosomes and bioactivity of the drug. However, continuous upgradation and optimization are carried out to arrive at a suitable methodology and conditions for drug loading process, as exosomes mediated treatment can be much easier, cost effective and provide good efficacy against the disease condition due to high drug loading concentrations [157,107].

### 3.3 Drug delivery applications

Site targeting is one of the important steps in exosome mediated therapy after successful loading of the therapeutic components. Primary sites of accumulation of exosomes are reported to be liver, kidney, and spleen. However, researchers are modifying the core and surface of exosomes for increased accumulation at the specific site of target [112]. Lipid and proteins molecules on the surface are the primary point of contact between the exosomes and donor/recipient cells. Surface molecules interact with receptors in the target cells through natural interactions [97]. Delivery of mesenchymal cells derived miRNA-124 to glioblastoma cells, MDA-PCa2b (prostate cancer cells) derived miRNA-141–3p to osteoblasts were successfully achieved through exosome-dependent pathway [109,110]. Exosomes from tumor-associated fibroblasts possess several integrins in their surface such as integrin αVβ5 target liver cells while integrin α6β1 target lung cells [170]. This provides excellent opportunities to exosomes mediated cancer therapy as exosomes from single type of cells has ability to interact with different cell types. Though drug molecules can be distributed to different part of the system through exosomes, it is undeniable that they are also a potential source of molecules that aids in metastasis. Hence, strategies to avoid these pro-cancerous molecules from the exosomal core or surface is an important point to be addressed [33,157,118].

Surface functionalization of exosomes with target ligand molecules that can potentially interact with recipient cell surface receptors is another valid strategy for a successful exosome mediated drug delivery. Lactadherin C1C2 domain and Lamp2b with synthetic tags are some of the commonly studied targeting proteins in the exosomal surface [171]. αV integrins are one of the conserved membrane proteins expressed in most of the tumor cells [119]. Cyclic peptide, iRGD in malignant tumor has high affinity to the surface integrins of malignant tumors and a study involving the over expression of iRDG and Lamp2b on the surface of the immature dendritic cell (imDC) derived exosomes showed significant targeting to the breast cancer cells [172]. Doxorubicin loading into the imDC derived exosomal core has shown significant efficacy against breast cancer cells [120]. Similarly, brain targeting was successful with exosomes containing rabies virus glycoprotein (RGV) and related peptides; c(RGDyK). Delivery of miRNA-124 was successfully carried out for brain infarction treatments when exosome surface was modified by RGV fusion with Lamp2b protein [173]. Treatment for morphine addiction was carried out by delivering morphine receptor silencing siRNA to the Neuro2A cells or mouse brain through exosomes containing RGV proteins on the surface [174]. Binding ability of Klotho protein to the surface of endothelial progenitor cells have provided new path for exosomal mediated delivery for certain endothelial therapeutics. Transfection of Klotho gene into the mesenchymal cells and obtaining exosomes loaded with adenosine kinase siRNA by electroporation showed significant delivery of the drug components to the endothelial cells [111]. Another strategy of specific targeting ability of exosomes was developed with nanoparticle-exosome combination by membrane modification in the donor cells or exosomes. Nanoparticles that stabilize the glycosylphosphatidyl inositol (GPI) on the exosomal surface showed very good presentation of reporter proteins, signaling molecules or immunomodulating antibodies [112].

Surface coating of exosomes with antibodies were developed for many cancers therapeutic applications. A33 positive exosomes were isolated from LIM1215 ​cells and doxorubicin was included in the exosomal core. Further, surface coating of A33 antibodies with iron oxide nanoparticles showed significant inhibition of cancer growth [113]. Infrared mediated nanoparticles like gold nanorods, when coated on to the surface of exosomes and irradiated with light, increase in temperature with increased permeability of exosomes was observed aiding in release of drug molecules into the site of interest [121]. Aminoethyl-anisamide-polyethylene glycol ably targets the lung cancer cells and is used to coat the exosomal surface that was added with paclitaxel showed high drug loading capacity and accumulation in the cancer cells [114]. Antibodies-like RNA was reported to possess high affinity towards surface markers of prostate cancer, breast cancer or colorectal cancer cells. Hence, loading small molecule inhibitors or siRNA to exosomes with antibodies-like RNA showed significant cancer inhibition without observable toxicity [115]. Glycoprotein based adhesion molecules on platelet surface helped in targeting and retention of drug in the damaged tissues. Platelet nanoparticles was successful in guiding several stem cells and pertaining exosomes to mediate cardiac tissue repair [116]. Apart from triggering the expression of desired molecules and natural mechanism of exosome sorting, recently the functionalization of exosomes after isolation has gained significant interest like conjugation of PEG with phospholipid derivative to target the epidermal growth factor receptors. This nano conjugation of phospholipid has significantly improved the systemic circulation of the engineered exosomes and specifically targeted the tissues of interest [117].

The theranostic applications of exosomes discussed above, helped in management of several diseases uniquely. However, their clinical translation still in its early stages, because of the issues pertaining to quality and quantity of the isolated exosomes. There are a lot of unexplored territory in biological process of exosome generation, which is a huge road block for the clinical translation. Complete understanding of the biology of exosome at the cellular level may throw light on the molecular and biochemical profile and will help in manipulating the exosome generation pathways, engineering exosomes and exosomal targeting strategies, according to the requirements. The pharmacological stability is another important aspect to be considered for the development of exosomes-based therapeutics. Quality of purified exosomes are greatly affected by other EV components that significantly affects the physico-chemcial properties of exosomes and in turn the efficacy of the application. Significant research and development on optimizing the isolation processes are being conducted to fill the gaps in theranostic applications of exosomes due to quality and quantity of exosomes. The isolation methodologies and challenges faced during each methodology were discussed in the following section, which clearly states that continuous exploration is absolutely required for optimizing the exosome isolation protocols.

## 4 Isolation methodologies and challenges

**Fig. 2:**
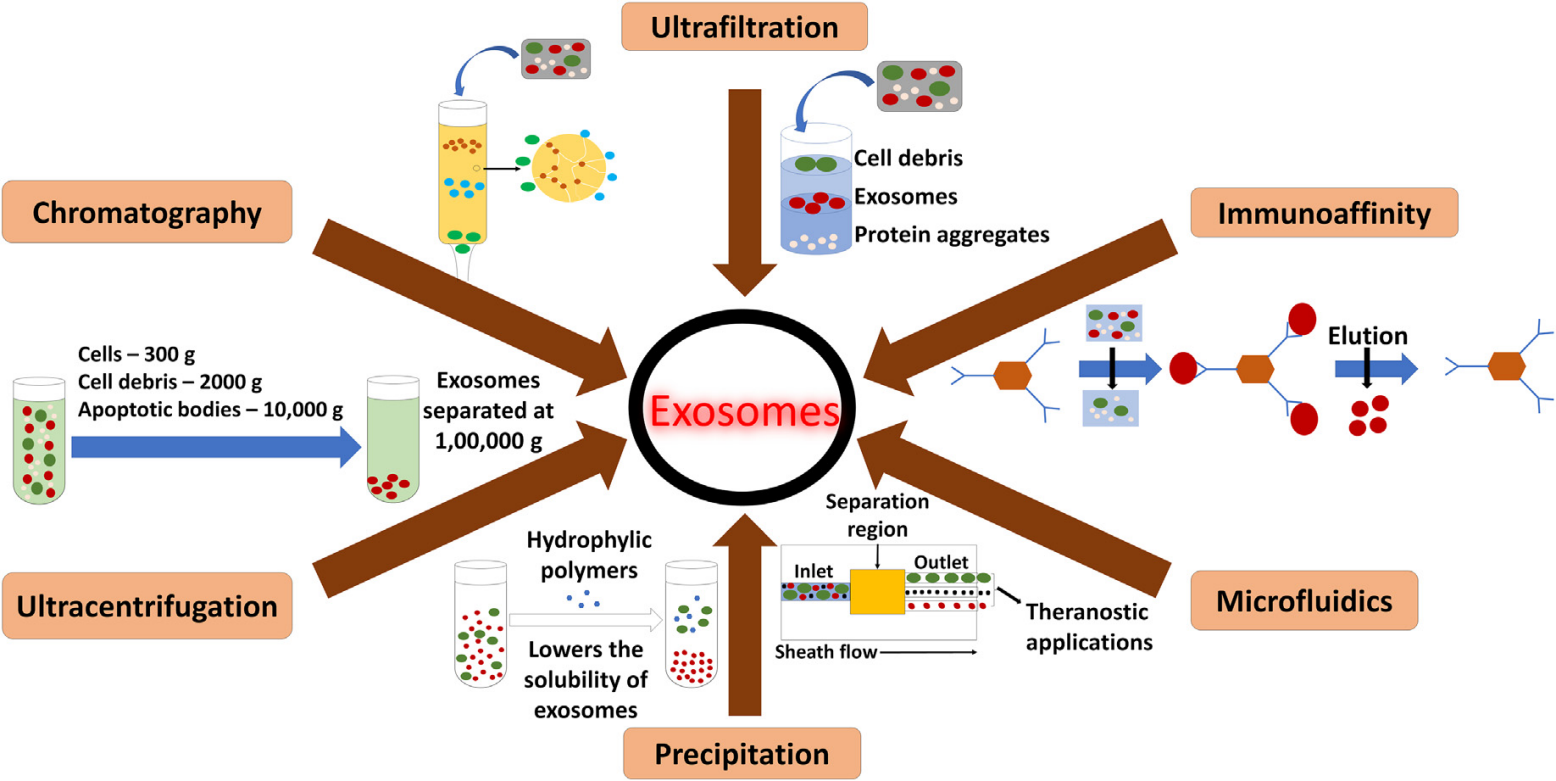
Various isolation methodologies of exosomes [134].

The first and crucial step during any of the above-mentioned applications is isolation of exosomes from the donor cells. Important aspects which should be considered during development of an isolation protocol are finding the methodology with optimal conditions that does not affect the overall biological functions of the exosomes, biochemical profile of the exosomes, structural integrity of the exosomes and finally aid in increased yield of exosomes with less impurities [122]. The exosomes are to be separated out from varied size of particles in the form of virus, bacteria, intracellular organelles, and other extracellular vesicles that includes microvesicles and apoptotic bodies [126]. Many methods are employed for successful isolation of exosomes but most of them are broadly grouped into 6 major categories based on their working principle (Fig. 2). Isolation of exosomes were achieved by ultracentrifugation based on the size and density, whereas ultrafiltration and chromatography (especially gel-filtration chromatography) exploited the size of the sample based on which exosomes were purified from the other cellular components. Immune-affinity based isolation methods involves the specificity of surface proteins, polymer precipitation involving the difference in solubility and microfluidics-based isolation technologies.

Isolation methodologies have shown significant success and aided in exosome-based therapeutics. Though several commercial kits were developed based on the separation methodologies, the drawbacks in each method are still highlighted and current research studies were focused primarily on filling the gaps in separation methods [123]. One of the major disadvantages in all the methodologies, is the purity of the resultant exosome fraction. Hence, combining the separation methods to obtain a hybrid methodology helps in enhancing the purity of the isolated exosomes [124]. Ultracentrifugation is relatively easy method to perform and despite several advantages, the vesicles of varied sizes ranging from 50 to 1000 ​nm were observed. This heterogeneity in size leads to overlapping of the vesicles and contamination is a common issue [123,175]. Density gradient ultracentrifugation has concerns with exosomes of low density floating at the top layer, which require another round of centrifugation [176]. In case of ultrafiltration, stationary phase obstruction by microvesicles make the exosomes to be trapped in between. This reduces the efficiency of the membrane or stationary phase with number of separation procedure [177,178]. The instrumentation used for size exclusion procedure is little complex compared to other methodology and its run time is also very long. In case of immunoaffinity capture methodology, low yield, and chance of false negative outcome due to heterogeneity in tumor antigen are some of the disadvantages [179]. The exosome precipitation method has a problem with isolation of specific exosomes and low purity. Several other aggregates like protein, micro vesicles and etc., from body fluids interfere with the purity of isolated exosomes. Further, the complex biomolecular profile of the body fluids leads to difference in viscosity and the sample contents other than biomolecules makes the optimization of the precipitation methodology meticulous [124]. Microfluidic approach is still had not succeeded in clinical translation mainly due to scaling up for the large sample numbers apart from absence of pre-clinical validation and standardization [180]. The sample treatment before application into the device was more laborious and exosomal loss during the pretreatment protocol leads to low quantity of recovery. This has significant effect on the downstream isolation and analysis of the biomolecules which are reported to be very poor [126,178]. Fusion of two different methods improved the purity and quantity of isolated exosomes. However, the cost included in the development process is very high and strenuous training is required for understanding the working knowledge of the instrumentation. Increased sample preparation and isolation steps are generally prone to instrumental and human errors [175,181,182]. To overcome these drawbacks and other practical issues, continuous research and development is required. One of the widely accepted hypotheses is the influence of external energy in improvement of isolation efficiency and quality of the isolated exosomes [183,184]. Ultrasound assisted exosome isolation is particularly advantageous over other protocols and advancement in ultrasound technology has opened several gates in exosome research. Acoustic waves have been mainly incorporated with microfluidics devices and aid in isolation step, while it was also helpful in increased exosomal release from the cells [184,185].

## 5 Research and development in ultrasound-based applications in biology medicine

Clinical imaging is one of the successful tools in diagnosis and prognosis of various complex diseases. Image based diagnosis was an integral part of management of cancer, neurodegenerative diseases and even provide valuable information regarding the progression of certain psychological disorders [186,187]. Among the imaging modalities Magnetic resonance imaging (MRI), Computed tomography (CT) and Ultrasound provide significant insights on the disease status with minimal invasiveness [188]. Conclusive evidences were provided by these imaging methods without any contrast enhancing agents, hence clinical diagnosis and decisions regarding therapeutic management are successful most of the times [189]. However, there are some exceptions, where contrast agents are needed to differentiate overlapping symptoms of different pathological conditions in the same organ or to define the disease stages [190,191]. Ultrasound is one of the widely used clinical imaging techniques which work under echo imaging principle [192]. The instrumentation consists of a transducer that generate acoustic waves, the echo receiver, beam former, digital converter that converts the signals to data (ADC) and finally computer-based data and image processing system [193]. The transducers are made of piezoelectric material that generate ultrasound waves in 1–15 ​MHz range, which propagate through tissues and echo signals after the tissue interference were received. Change in acoustic impedance in the echo signals were received back to the transducer which were processed by ADC. The converted signals were focused by the beam former for a better image quality. The images were acquired in B-mode (Brightness), M-mode (Motion) and Doppler mode (Blood flow characteristics) [194].

Ultrasound based imaging method was considered for both diagnosis and guided therapy which was successful against many diseases which are summarized in Table 3. It is commonly preferred over other imaging methods because it is inexpensive and easily portable. The instrumentation is suitable for easy handling, and it is non-invasive. Poor penetration into air filled tissues or bone makes it not suitable for imaging lungs, brain, and abdomen. Though there are some significant drawbacks reported against ultrasound, the preference over other methods was given by clinicians because of it safe and cost-effective methodology [195]. Ultrasound has been playing key roles in several research field other than medicine. They are used in decontaminating the food products from pesticides such as chlorpyrifos, methamidophos, τ-fluvalinate, malathion etc. Milk products were processed through ultrasound methodology to remove food allergens like lactoglobulins, tropomyosin, casein etc., [196]. Cui and Zhu have organized several reports into a review where they have included several possible polysaccharides, whose efficacy has improved significantly by ultrasound irradiation with major changes in their physico-chemical properties [197]. Apart from this, ultrasound has become an integral part of food processing operation and medicinal plants extraction methodologies to obtain bioactive compounds [198]. Ultrasound assisted enhanced germination of cereals were comprehensively discussed by Wang et al., 2019, where the ultrasound-based techniques not only helped in germination process but also influenced the crop resistance against several environmental stresses, at molecular level [199]. Further several studies have discussed on the ability of ultrasound in preparation of more safe and efficient fertilizers that aid is germination and growth of different crops and on the other hand ultrasound-based methods have been successful in water treatment to remove organic and inorganic contaminants [200,201]. It is not just the diagnostic property of the ultrasound that is utilized in the clinical sector, but the acoustic property is being utilized and has succeeded in preparation of the active pharmaceutical ingredients as stable nano/micro formulation [202], targeted delivery of the drugs [193], improvement of the pharmacokinetic property and reducing the toxic property of drugs [203]. However, the therapeutic application of ultrasound was achieved with significant changes in the experimental parameters. During diagnostic imaging ultrasound intensities were maintain at a range of 0.05–0.5 ​W/cm^2^. Therapeutic ultrasound applications work mainly with the thermal and mechanical effects of acoustic waves and hence, high intensities from 0.2 ​W/cm^2^ and up to 10,000 ​W/cm^2^ was used. Major difference between the imaging and therapy based on ultrasound is that, imaging method captures the acoustic signals from the tissues as echo and theory behind therapeutic ultrasound is the biophysical effects of ultrasound like thermal and mechanical effects [204].

**Table 3:** Theranostic applications of ultrasound in clinics and nanomedicine.

Type of application	Methodology utilized	Disease condition/process influenced	Reference
Neurological applications	Thermal ablation with high intensity ultrasound (1–3 ​MHz); Mechanical effect with medium intensity (>100 ​kHz); non-thermal effect (<100 ​kHz). Brain ablation with focused ultrasound	Parkinson's, essential tremors, pain, neuropsychiatric conditions	[205]
	MR guided focused ultrasound	Essential tremors	[206,207]
	MR-guided pallidotomy with FUS	Parkinson's disease	[208]
	Focused ultrasound	Crossing blood brain barrier	[209]
	Focused ultrasound	Neurovascular conditions	[210]
	MR guided focused ultrasound	Angiogenesis during cerebral hemorrhagic stroke	
Cardiovasular applications	Ultrasound in B-mode	Characterization vascular muscle walls	[211]
	Ultrasound in M-mode	Blood vessel movements	[212]
	Doppler mode	Blood flow characteristics	[213]
	Phospholipid-based microbubbles enhanced ultrasound	Endocardial imaging	[214]
	ICAM-1 mediated targeting of endothelial cells	Ischemia	[215]
	P-selectin	Cardiac imaging	[216]
	Microbubble loaded VCAM-1, ICAM-1 and P-selectin	Atherosclerosis	[217]
	Fibrin targeted microbubble	Thromboembolism and acute coronary syndrome	[218]
	αIIbβ3 complex co-loaded with urokinase	Delivery to site of thrombus	[219]
	Microbubble loaded with oxygen dependent prolyl hydroxylase (PHD2) and matrix metalloproteinases 2 (MMP2)	Improvement in myocardial function and decrease in infarct	[220]
Hepatic complications	Conventional US	Parenchymal morphological analysis, possible fibrosis, cirrhosis or hepatocellular carcinoma and portal hypertension	[221]
	Conventional US and sonographic hepatorenal index	Steatosis	[222]
	Acoustic structure quantification (ASQ)	Differentiation of fibrosis and steatosis	[223]
Renal complications	Conventional US	Chronic kidney disease diagnosis	[224]
	Conventional US	Renal lithiasis	[225]
	Color Doppler analyses	Blood flow characteristics and renal parenchymal perfusion	[226]
	Doppler studies	Blood velocity and alterations in renal vasculature, Resistive index	[227]
	Resistive index analysis	Renal vasculitis, Glomerulo/Lupus nephritis, diabetic neprhopathy	[228]
	Contrast enhanced ultrasound	Tubulointerstitial injury	[229]
Cancer	Conventional US (intraoperative imaging)	Neurosurgery, Brain tumor resection surgery and management	[230]
	Conventional US	Differentiation of malignant from normal/benign tissue	[231]
	Conventional US	Management of thyroid cancer, ovarian cancer (Differentiation of cystic composition and cancerous lesions in ovary),	[[232], [233], [234]]
	Conventional US	Prostate cancer diagnosis	[235]
	High intensity FUS	Treatment of urethral strictures or epididymitis during prostate cancer	[236]
	High intensity FUS and MRI guided HIFU	Tumor ablation	[[237], [238], [239]]
	HIFU	Liver tumor removal	[240]
	MR guided US	Bone metastases	[241]
Nanoformulation	Microbubble and ultrasound (Interior gas phase mediated and exterior liquid phase mediated)	Nanoparticle synthesis	[242]
	Ultrasound irradiation, cavitation of microbubbles	Nanoemulsification, exfoliation of multilayered particles	[243]
	Ultrasound treatment	Herceptin loaded graphene	[244]
	Ultrasound guided chemical co-precipitation	Hydroxyapatite/TiO2 nanocomposites	[245]
	Laser pyrolysis combined with ultrasonic nebulizer	Zinc containing nanoparticles or nanocomposites (ZnS, ZnF or ZnO)	[246]
	Ultrasound induced oxygen radical generation	Bovine serum albumin crosslinked microspheres	[247]
	Chemical effects of ultrasound	Plant-based oil and wheat germ agglutinin, starch-based nanoparticles	[248]
	ultrasonication	Superparamagnetic iron oxide nanoparticles containing nanocomposites composed of polymeric methyl methacrylate	[249]
Therapy	Sonosensitization	Chemotherapeutic drugs and non-steroids anti-inflammatory drugs, porphyrins compounds, pyrrole derivatives, 5-aminolevulinic acid, cholrin E6, methylene blue	[250]
	Ultrasonication	BBB permeability	[251]
	Ultrasonication mediated ROS generation	Glioma treatment	
	Sonodynamic therapy combined with immune and hormonal therapy	Breast cancer treatment	[252]
	Sonodynamic therapy	Liquid tumors	[253]
	Sonodynamic therapy mediated delivery of cyclosporin B	Actin inhibition and reduction of tumor growth	
	Sonodynamic therapy	Anti-microbial therapy and periodontitis	[254]
	Sonodynamic therapy	Treatment of atherosclerotic plaque	[255]
Drug delivery	Pluronic P123/F127 loaded with curcumin	4T1 breast cancer cell delivery and inhibition	[256]
	DOX loaded PLA microbubbles; surface coated with TRAIL	MDA-MB-231 & MCF-7 cells-based tumor growth by site targeted delivery	[257]
	Gemcitabine encapsulated PLA bubble	Pancreatic cancer delivery	[258]
	Mesoporous silica nanoparticles loaded with plasmid DNA encapsulated in polymeric shell	Ovarian cancer delivery	[259]
	Loading O_2_ loaded microbubble coated with Span 60 and vitamin E	Oxygenation of breast cancer and making it radiosensitive	[260]
	Lipid-based microbubbles coated with chemotherapeutic drug encapsulated with perfluorobutane	Drug release and inhibition of tumor growth	[261]
	DOX-loaded liposomes coated with magnetic iron oxide nanoparticles were encapsulated with perfluorooctane	Drug release and inhibition of tumor growth	[262]
	Sonoporation with Optison, Definity, Lumason/SonoVue and Sonazoid	Deeper penetration of drugs	[263]

### 5.1 Ultrasound & clinics

The applicability of ultrasound was recognized and brought into light in 1950s and this started with John William Strutt (Lord Rayleigh), who clearly described the theory of ultrasound and its applicability in various field [[264], [265], [266]]. Diagnosis of mitral stenosis by echocardiogram and abdominal imaging with pulsed ultrasound has revolutionized the medical imaging [9]. Despite the initial breakthroughs, issues like heavy instrumentation, failure to provide appreciable image quality, etc., were reported and this led to significant research focus on developing a better performing, portable instrumentation with advanced algorithms to acquire images with high quality [[267], [268], [269]]. Ultrasound has become a routine imaging method in clinics and has proved to be effective in many pathological conditions like fibrosis [270], abnormalities in abdominal organs [271], obstetrics [272], etc. However, sometimes clinicians rely on ultrasound to obtain conclusive evidence along with other imaging modalities due to complexity of the pathological condition or severity of the disease stage [273].

#### 5.1.1 Neurological applications

Neurological applications of ultrasound have gained significant improvement recently after its ability to aid in neurosurgical procedures for essential tremors, Parkinson's disease, neuropathic pain and certain neuropsychiatric conditions like depression, anxiety and obsessive-compulsive disorder [274]. These are certain thermal ablation-based applications where higher intensity of ultrasound (>100 ​kHz; 1–3 ​mHz) were used, while medium (>100 ​kHz) or low (<100 ​kHz) intensity ultrasound can induce mechanical and non-thermal effects in the tissues, respectively [275]. Although poor penetration through the skull for neuroimaging has limited the use of acoustic-based therapies, while recent developments showed real-time monitoring of ablation was possible through Magnetic Resonance (MR) guided focused ultrasound. This is a non-invasive procedure that avoids surgical procedures like incision or electrode penetration and is also approved in many countries [276]. Apart from this, MR guided FUS thalamotomy is frequently advised by experts for essential tremors when the patients did not respond to medications. Studies showed significant recovery in the patients with reduction in tremors and improved motor ability. However, the long-term adverse effect was observed in about 1/3rd of the patients [277,278]. Ability of ultrasound-based therapy to treat essential tremors has led the researcher to conduct MR-guided pallidotomy with FUS, which was a new advancement in this field, where the FUS is focused mainly targeted at the excitatory nerve fibers at pallidum connecting to the thalamus (Pallidothalamic tract) [279]. This could lead way to a better treatment strategy to Parkinson's disease patients who are medically refractive [205]. However, the best target location in the brain for treatment of Parkinson's disease is not identified yet and researchers are focusing on this regard to narrow down to a common target area or consider this as patient dependent [280].

Ultrasound has also shown potential in improving the pharmaceutical properties of therapeutic drugs through efficient drug delivery across the blood brain barrier (BBB), also in drug sensitization using low-intensity acoustic waves at the target site and further help in drug internalization by sonoporation process [206]. Facilitating a drug to cross the BBB is one of the unsolved struggles in pharmacokinetic research and FUS has shown significant promise in this regard with transient opening of BBB through cavitation and microbubble injection [207,281]. Recently, treatments involving sonoporation for drug internalization and sonosensitization for activation of drugs were performed together and it is termed as sonodynamic therapy [208]. This could be achieved with low-intensity ultrasound that produce non-thermal and mechanical effects on the tissues [282]. Similarly, ultrasound has shown its potential in influencing certain intracellular processes for modulating neural activity, inhibiting inflammatory signaling, angiogenesis and tissue regeneration. Neuromodulation can also be achieved by applying low-intensity pulsed US and this can directly influence the neural activity, or a drug molecule can also be facilitated through the BBB by applying FUS. These US-based methods provide reversible/irreversible neuromodulation without any thermal ablation [283,284]. Neurovascular applications of US have been well established and several clinical trials were performed to test the effectiveness of US in thrombolysis, recanalization, functional improvement of the vessels [285]. [286]. However, the application of US alone did not provide expected outcome and hence, it is utilized as an adjunct to tissue plasminogen activator (tPA) based therapy [287,209]. Angiogenesis during cerebral hemorrhagic stroke, was reported to be successful with MR guided FUS and though sonothrombolysis along with angiogenesis was also effective and showed great promise, further explorations are required to reduce the side effects [288,210].

#### 5.1.2 Cardiovascular applications

Ultrasound is one of the frequently preferred diagnostic techniques for cardiovascular conditions as it does not cause any discomfort to the patient and has no risk of radiation and so, it can help in a monitoring the progress of the disease condition and provide a comprehensive observation about a patient that can help in identifying suitable treatment method [289]. Vascular muscle walls were characterized in B-mode [290], while pulse wave and Doppler mode were used to study the blood flow characteristics [291,292]. Myocardial motions and blood vessel movements were recorded in M-mode [293]. Apart from this several other methods like strain imaging, contrast echocardiography, point of care ultrasound (POCUS), 3D echocardiography, 3D volume flow measurement and elastography were also used for imaging cardiovascular system for various vascular complications. MRI and CT coupled angiography are the imaging modalities still ahead of US imaging without contrast enhancement, which fails 10–15% of the times due to poor delineation of endocardial border [294]. This may be because of several drawbacks in the ultrasound-based imaging methods. Doppler imaging has several disadvantages like longer acquisition period, complicated post processing of images and relatively low reproducibility. The strain imaging based on doppler effect highly depends on the angle of image acquisition to obtain high frame rates, while 3D echocardiography suffers from low spatio-temporal resolution. POCUS is not a comprehensive imaging technique and cannot provide complete pathological information but is effective in providing rapid clinical care [211]. All the ultrasound-based methodologies suffer from shortcomings and researchers are constantly working towards addressing these issues or improving the instrumentation for getting images with better quality. One of the important findings for enhancing the quality of ultrasound images, is the discovery of contrast agents like phospholipid-based microbubbles filled with gases like octafluoropropane, perfluoro propane etc. [289]. There is a marked difference in the acoustic impedance between the gas encapsulated microbubble and the nearby tissues, which leads to significant increase in the backscattering US signals [212].

Tissue perfusion imaging and endocardial border delineation are the major imaging methods being practiced in the clinics and these are highly recommended methods which helps in occlusive and pre-occlusive stenosis, blood flow characteristics of the repaired vessels and physical characteristics of the plaques [213,295]. Chance of false diagnosis due to overlapping symptoms has directed the researchers and clinicians to rely on molecular contrast agent guided US. It is very successful in precise diagnosis for myocardial infarction where the microbubbles are targeted towards leucocytes through complement system or leucocyte specific ligands coated on the surface of the microbubbles [[214], [296], [297]]. Detection of ischemia through intercellular adhesion molecule-1 (ICAM-1) mediated targeting of endothelial cells, P-selectin targeted detection are some of the advancements in the field of cardiac ultrasound technology over the conventional reperfusion-based methods [298,299].

Atherosclerosis was also showed to be effectively imaged using targeted microbubble mediated ultrasound [300]. Recently Yan and co-workers developed a microbubble targeting 3 markers (VCAM-1, ICAM-1 and P-selectin) which are the key players in atherosclerosis has given a new dimension to management of atherosclerosis [301]. Thromboembolism is a characteristic feature of acute coronary syndrome and fibrin targeted microbubble helps in locating the thrombi at different parts of the system. Further to avoid cross reactivity with fibrinogen, researchers have targeted activated platelets using αIIbβ3 complex, while urokinase co-loading has helped in targeted delivery to the site of thrombus [215,216]. Current revascularization-based treatments for stroke are performed with thrombolytic drugs where the dosage and duration of treatment depends on location and size of occlusion. Increased and prolonged dosage could lead to hemorrhage and other pertaining adverse effects. Hence, US was utilized for thrombolysis by microbubble based mechanical dissolution. Facilitating the thrombolytic drugs deep into the thrombus has shown significant success in pre-clinical and phase II clinical trials. The microbubble-based US application has shown significant promise in controlled delivery of various drug molecules [302,217]. Marked development in gene therapy for cardiovascular complications was observed recently, despite its pharmaceutical limitations. Microbubble loaded with oxygen dependent prolyl hydroxylase (PHD2) and matrix metalloproteinases 2 (MMP2) has shown to improve myocardial function and decreased the infarct [218,219]. Continuous explorations are underway to address the limitations like inflammatory reactions, hemolysis with in heart, microvascular rupture and leakage [303].

#### 5.1.3 Hepatic and renal conditions

Morphological level alteration in the kidney is well observed in B-mode US imaging and color Doppler or contrast enhanced ultrasound (CEUS) provide changes in the organ perfusion [304]. Conventional US also helps to assess the liver parenchyma for any lesion that can lead to possible fibrosis, cirrhosis or hepatocellular carcinoma and portal hypertension. SD elastography have been successful in deciphering different stages of liver fibrosis [220].

Reduction of longitudinal renal diameter is the hallmark of chronic kidney diseases leading to significant reduction in the glomerular filtration rate [305]. The parenchymal thickness is another important feature to be noted during CKD diagnosis, which is the distance of capsule and the base of the renal pyramids while performing the longitudinal scanning of kidneys [306]. The color Doppler analyses provide valuable information for other diagnostic techniques, while spectral Doppler studies provide valuable information on blood velocity and the alterations in the renal vasculature [307]. A shift in Doppler frequencies provide information about the blood flow characteristics and measures the resistive index (RI). It is the sum of resistance forces that opposes the arterial blood flow and gives an assessment about the microcirculation in the renal system [221].

Most of the renal complications were diagnosed and the severity was also given with RI value. Normal kidney has the RI of approximately 0.6, 0.7 is considered as the upper limit, above which the kidney can be categorized as CKD [308]. Glomerulonephritis and tubulointerstitial atropy are the common feature of almost all the renal pathological conditions at the advanced stage of CKD and hence, RI was reported to reach up to 0.7 and stage 5 CKD showed the RI value of 0.8 [224]. The change in RI value and image-based evidences for the pathological changes in the kidney provides precise and conclusive information about the disease status which helps in deciding the optimal treatment strategy to the patients [226]. Certain conditions like secondary nephropathies accompanied with glomerular damage, shows RI more than 0.75 to 0.8 because of the co-existing microvascular impairment due to vasculitis, glomerulo/lupus nephritis [307]. On the other hand, the diabetic nephropathy (DN) with characterized microalbuminuria, hyperfiltration and uncontrolled glycosylated hemoglobin was detected with low RI (∼0.5) and high GFR [227]. But, in later stages of DN the RI significantly increases due to microvascular injury and reduction in GFR [228]. Tubulointerstitial injuries are very difficult to diagnose using US due to its complexity and combined observation from a second level imaging technique or CEUS are required to derive the exact clinical conditions [229]. In case of urinary calculi or any mode of urinary obstruction, US is the first imaging technique employed for diagnosis and they are observed as hyperechoic and forms an acoustic shadow [309]. US was reported to be 95% successful with renal lithasis but only 35% accurate in case of ureteral lithiasis. Secondary complexities due to urinary obstruction such as hydronephrosis, ureteral dilation or bladder stasis are very well diagnosed using US with 100% sensitivity [310]. Though a skilled nephrologist can diagnose up to 70% of the CKD patients, at advance stages of renal damage with reduced kidney size and unstructured morphology, US based detection and accurate diagnosis with staging, of a particular condition is very difficult [311].

Similarly, the change in morphology of the hepatic tissues and convincing pathological observations were reported with US. Conventional B-mode imaging proved a qualitative score of the liver parenchymal morphology, where diaphragm and hepatic blood vessels cannot be seen distinctly in a fatty liver condition compared to normal liver imaging [220,312]. Steatosis detection at early stages varies with inter-person imaging and liver brightness holds the key to an effective diagnosis. Hence, sonographic hepatorenal index was introduced, where segment 6 of liver and upper pole of right kidney were visualized and the brightness was measured in pixels at the region of interest. The SHRI stands for the ratio of mean liver to renal cortex brightness [313,225]. The correlation between the histological findings and SHRI were reported in several studies and its ability to diagnose mild steatosis shows its success among the other grading methods. Despite the success of SHRI grading system, optimization of the technique is still required to avoid interobserver variation [314]. Another feature of ultrasound that helps in differentiating the steatosis and fibrosis is speckle noise that occurs as scatters due to interferences during imaging. Acoustic structure quantification (ASQ) is a type of scoring system used to differentiate fibrosis and steatosis using the statistical deviation in the ultrasound signals [315,222].

#### 5.1.4 Cancer diagnosis and therapy

Outcome of constant exploration for cancer management strategies led to development of several interdisciplinary approaches in every stage from diagnosis, drug delivery, treatment, and long-term prognosis. Cancer management has been approached through physical, chemical, and biological methods. Recently after the success of material science and nanotechnology applications in the medicine, clinicians started embracing the interdisciplinary approach [316]. Among the well-established interdisciplinary strategies for diagnosis and treatment of cancer, image guided methods and chemo-sensitization using the biomedical instrumentations has shown tremendous success [317]. Ultrasound is part of the clinical imaging method with great success with respect to cancer management over other successful major imaging methodologies. This could be because of the absence of ionizing radiation, easy portability, and real time visualization through the US-based method [223]. Halliwell in 2010, has reported that among the diagnostic images generated, 20% of them are US-based images [318].

Ultrasound has been successful in brain tumor management, especially US has been an essential part of brain tumor resection surgery. Intraoperative US imaging is very helpful for brain tumor surgery mainly for localization of tumor and delineating the margins without affecting the flow of the surgery [319]. Brain tumor treatment by *trans*-cranial MR guided focused ultrasound was considered as a better option than other successful treatment strategies. Neurosurgery for maintenance of brain function was successful in many clinical studies with reduction in pain [320,321]. Diagnosis of breast cancer has been successful with the help of ultrasound, which provides better differentiation of malignant from normal/benign tissue in comparison to commonly preferred mammogram-based diagnosis [322,230]. For any disease condition, it is necessary to diagnose the stage of the disease early and accurately for providing a suitable and timely treatment. Ultrasound has significantly aided the diagnostic imaging of cancer specifically, up to 17% increase in clear and accurate diagnosis. This has led to almost 40% decrease in the number of biopsy testing, for cancer diagnosis [323]. US has also shown its success in accurate diagnosis and management of thyroid cancer. Fine needle aspiration-based biopsy helps in diagnosis of thyroid cancer and US has been instrumental in guiding the technicians to locate the region of interest for fine needle aspiration (FNA) biopsy sampling [324]. Similarly, the US has been very effective in diagnosis of ovarian cancer lesion and distinguished them clearly from normal or benign tumors septum [231]. Morphological features such as cystic composition or solid tumor lesion, of ovarian tissue and pertaining cancer lesions were observed through US imaging with internal echoes occurring irregularly and septum formation that are considered as the gold standard in ovarian cancer diagnosis. As discussed previously, scoring system is an integral part of US methodology in several diseases, especially the cancer diagnosis [[325], [326], [327]]. However, the limitations like unsuitable for clinical practice without proper scoring system were constantly addressed [323]. Prostate cancer diagnosis was either performed digital rectal examination and further tissue biopsy was performed after a transrectal ultrasound imaging-based confirmation [328,232]. Some drawbacks like limited availability of clinical protocols and difficulty in imaging during edema conditions were reported. Inability to set a theoretical baseline/cut-off value is another concern with US, despite having scoring system as an integral part of diagnosis [233].

Therapeutic application of ultrasound and acoustic systems have been well exploited for cancer management. The use of focused ultrasound technique for experimental study and therapeutic trial was first held in 1942 and then it was well established in 1970. Tumor ablation was found to be possible when focused ultrasound was applied at high intensity, where in FUS at a higher frequency was applied to manipulate the mechanical effects such as cavitation, microstreaming etc., and thermal effect causing elevation in temperature up to 60 ​°C, for tumor removal [233]. In case study of prostate cancer treatment with HIFU (Sonablate® device), the overall survival rate of 918 patients in the study showed up to 89.6% and about 97% of overall survival rate for a period of 10 years. Though there were notable side effects due to HIFU treatment reported in this study, such as, urinary inconsistency caused by urethral strictures or epididymitis, it showed promising evidences as a better therapy for prostate cancer [234]. Therapeutic management of breast cancer has improved significantly over the years and currently breast conserving therapy is adopted to avoid mastectomy. However, treatment for early breast cancer, non-invasive ablation is considered as an alternative therapy [329,235]. Results from many clinical studies denoted that up to 71% of patient showed complete tumor ablation, while 59% necrosis was observed with MRI guided HIFU and 96% with US guided HIFU therapy [[236], [330], [331]]. Reduced side effects such as absence of scaring, radiation, or bleeding apart from unchanged breast structure and function, during HIFU therapy are the major advantages of this method. Some drawbacks such as inability to identify if the ablation margin is free, chance of recurrence and remains of necrosis after therapy, are still needed to be addressed to make HIFU a common treatment method employed in the clinics [233].

Management of liver tumor was always challenging due to complexity in the etiopathogenesis, while ultrasound-based therapies were not considered as potential treatment modality because at present surgical removal of tumor or transplantation are considered as the best possible strategy [332]. However, ultrasound guided transcatheter chemoembolization was successful since, up to 45% of the patients showed complete ablation [237]. HIFU had major safety issues as it can lead to several complications like skin burns, pain, local damages like blockage of bile duct, rupture in diaphragm or damage to ribs, etc. Another significantly important drawback of HIFU based liver cancer treatment is chance for disruption of neighboring blood vessels [238]. Hence, in case of hepatic cancer management, ultrasound has been limited to intra-operative sessions to track the ablated zones, until optimal HIFU conditions were determined and clinical studies were conducted for short term and long-term treatments of advanced hepatocellular carcinoma [239]. Recent study with 187 patients with unresectable tumor reported that 90% of patients’ left lobe was cleared of the tumor, but only 64% of patients showed right lobe ablation [333]. Similarly, removal of renal cell carcinoma was performed using HIFU based ablation, cryoablation or radiofrequency guided ablation, only if the patients are unfit for the surgery [334]. Selective cases were also advised for extracorporeal or laparoscopic HIFU treatment to obtain a homogeneous ablation [335]. Primary and palliative pancreatic tumors with solid masses were also treated by HIFU based ablative procedure. After continuous research studies and trials, HIFU was found to be feasible and safer for locally advanced pancreatic cancer when performed in combination with chemotherapy [240]. HIFU has also shown its potential in treatment of bone malignancies with a response of up to 85% and patients also showed a reduction in pain with no device induced complications when the treatment carried out with MR guided methodology [336]. Gianfelice and co-workers showed MR guided US helped in significant removal of bone metastases with reduced co-morbidity [337].

### 5.2 Ultrasound & nanotechnology

Constant development in the field of drug discovery has led to identification of biological responsive materials that has the property to enhance the pharmaceutical stability and efficacy of the therapeutic drugs [316]. Biomaterials in nano/micro size range were constantly being explored for their biomedical applications as theranostic and drug delivery agent [338]. These drug incorporated biomaterials have also been sensitized and triggered by irradiating with external energy like light and acoustic waves at different intensity and wavelength range. Ultrasound and acoustic systems have shown its ability in sensitization of the biomaterials to help in internalization of the drugs by tweaking the cell dynamics and improving the pharmaceutical stability and efficacy of drugs. US also aids in synthesis of nanosized materials and mostly in combination with other methodologies to provide a material with better physico-chemical properties [339].

#### 5.2.1 Nano/microformulation processes

Ultrasound at high intensity were continuously being considered for nanomaterial synthesis as US helps in homogenization and heating up the precursors for production of small energy filled spots, which possess ability to drive chemical reactions. This excited nanotechnologists to develop US as an alternative for chemical based nano synthetic process, which require maintenance of optimal temperature and pressure for a longer time until the reaction is complete. US based material synthesis has gained significant interest and it possess diverse applicability [340,241]. It has clearly demonstrated its advantage over other synthetic methodologies especially in preparation of amorphous materials, mesoporous materials, and nanocomposites in which deposition of nanoparticles on to the ceramic or polymeric surface [341]. US irradiation first initiate cavitation that starts with accumulation of unwanted reactants and products into micron sized bubbles which finally collapse to induce various physical and chemical effects that helps in nanosynthesis process [342]. Nanomaterials composed of lipid derivatives and metal ions especially superparamagnetic nanoparticles were successfully synthesized using US based methodologies, where the chemical reaction for nanosynthesis was either triggered by gas-phase inside the bubble that collapses during US irradiation or in the liquid phase outside the collapsing bubble [343]. On the other hand, multi-layered material undergoing exfoliation, nano emulsification or nanoparticle surface modification are prepared by exploiting the physical effects of US [241]. Unstacking of multi-layered materials particularly graphene based nanosheets, is carried out by cavitation of bubbles that undergo drastic collapse generating shock waves with velocities around 4000 ​m/s [344]. Herceptin loaded graphene was reported to be stable and less toxic with improved efficacy, where graphene was processed and characterized by US treatment [345]. Generation of turbulence between two phases of immiscible liquids leads to formation of a new phase with fine droplets containing emulsion in sub-micron size. Major influencing factor in emulsification process is the ratio of oil to surfactant and water apart from the sonication time [242]. Several naturally occurring oil from medicinal plants and spices were prepared with surfactants where ultrasound played prominent role. Nanoemulsion made of cumin oil was prepared with Tween 80, dispersed using high intensity shock waves leading to formation of nanoemulsion with droplet size less than 150 ​nm [346]. Superparamagnetic iron oxide nanoparticles were prepared by chemical co-precipitation and the nanocomposites obtained from these particles were either prepared through mechanical stirring or ultrasonic waves. However, ultrasonic shock waves were able to produce stable nanocomposites compared to the mechanical stirring [243]. Synthesis of hydroxyapatite/TiO_2_ nanocomposites by chemical co-precipitation methods aided by US, resulted in nanosized (<20 ​nm) particles [244]. Nebulization by ultrasound produces droplets that moves with the surface of the liquid leading to atomizing effect. Fate of the droplets undergoing evaporation and drying leading to decomposition of reactants to form fine powders. Theoretically it can be achieved either by droplet to particle or gas to particle phenomenon [347,348]. Preparation of zinc containing nanoparticles or nanocomposites such as ZnS, ZnF or ZnO nanoparticles were prepared using laser pyrolysis combined with ultrasonic nebulizer [349]. Similarly, apatite particles were also prepared using ultrasonic vibrator mediated spraying method. Boron containing apatite particles were prepared using ultrasound mediated pyrolysis. Similar to calcium phosphate microspheres preparation, boron-based apatite nanoparticles were prepared but at a higher operating temperature (400–1000 ​°C) [245,350].

Apart from the nano synthesis methodologies exploiting the change in physical characteristics of the reactants under the influence of ultrasound, chemical characteristics influenced by ultrasound also mediate preparation of stable and effective nanoparticles [340]. Uni/multi lamellar liposomes, polymeric microspheres were reported to be synthesized either exclusively by sonochemical processes or in combination with the influence of physical properties of ultrasound [351]. Bovine serum albumin crosslinked microspheres induced by ultrasound induced oxygen radical generation, human serum albumin encapsulated in plant-based oil and wheat germ agglutinin, starch-based nanoparticles are some of the best examples of liposomes and microspheres prepared with the help of chemical effects of ultrasound [246,352]. Micro/nanogels sensitive to redox status and temperature was successfully prepared by sonochemical methods. BSA stabilized drug encapsulated in *n*-lauryl l-alanine methyl ester has shown noticeable stability to the formulation [353]. Similarly, preparation of Simvastatin encapsulated microspheres by ionic gelation process was aided by ultrasound, with optimal reactant concentrations and reaction parameters showed excellent mucoadhesive property and enhanced efficacy of Simvastatin [354]. US has also been considered as an inherent step in biological synthesis of metal-based nanoparticles such as silver, gold, zinc, etc. The studies reported an excellent improvement in the bioactivities of the nanoparticles. Nano-biocomposites composed of biologically important metal with magnetic property and organic components which were successfully prepared using ultrasonication process and is considered as an alternative method to thermal approach [340,241]. Similarly superparamagnetic iron oxide nanoparticles containing nanocomposites composed of polymeric methyl methacrylate was co-precipitated using Fe(II)/(III) with the help of ultrasonication [247]. Research works are being constantly conducted to identify optimal parameters to scale up the nanomaterial production process with the help of sonication.

#### 5.2.2 Sonodynamic therapy: sonosensitization, sonoporation and ultrasound assisted drug delivery

Sonodynamic therapy (SDT) has been a revolutionary development in the field of nano-biomedicine, where the physical phenomenon of sound was exploited to carry out chemical and biological processes. The concept of photodynamic therapy has led to the identification and development of sonodynamic therapy, where ability of sound to penetrate the tissues was utilized. The sensitization of drug molecules by activation of drug, providing accessibility to drug for cellular entry to facilitate certain biological mechanisms and aid in drug efficacy [248,355]. There are several mechanisms by which sonodynamic therapy was carried out. Some of them includes the well-known cavitation effect, generation of oxygen radicals, apoptotic pathway, immunomodulation against tumor. On the other hand, sono-sensitization does require certain specific compounds that can respond to acoustic waves and aid in potentiating the drugs molecules [355,356]. Responsive drug molecules include the chemotherapeutic drugs, non-steroids anti-inflammatory drugs, porphyrins group of compounds, pyrrole and derivatives, compounds like 5-aminolevulinic acid, cholrin E6, methylene blue, etc [355]. In most of the SDT, the frequency of acoustic waves ranges from 150 ​kHz–2 ​MHz and the exposure period were limited from 60 ​s to 30 ​min which vary according to the disease condition to be treated [356].

Significant research had gone into SDT for glioma and other neurological disorders, since one of the major challenges in neuropharmacology is facilitating drug molecules to cross the blood brain barrier (BBB), which can be achieved by either identification and preparation of molecules at optimal size that can cross BBB or utilizing some external physical or chemical force to facilitate the entry of drug molecules [248]. SDT performs the latter where, ultrasonic waves intermittently open up the BBB for drug molecules to enter the neuro system. SDT has reached up to clinical trial stages with respect to treatment of glioma which could be either direct effect of ultrasonic waves on tumor cells to undergo mechanical stress or ROS generation leading to apoptosis of the tumor cells [249]. Ultrasound based-breast cancer treatment was also well established and it was discussed in detail in the previous section. However, significant amount of exploration was carried out on SDT for breast cancer management and a study involved combination of sensitizer based SDT, immunotherapy and hormonal therapy showed significant reduction of tumor mass supported by downregulation of tumor markers. Though there were some manageable adverse effects observed due to SDT, the tumor reduction rate and patient survival rate has increased significantly [251]. Liquid tumors such as leukemia, lymphoma was treated well by SDT, by selectively targeting the tumor cells and inhibiting the actin production specifically in the tumor cells by supplementation of cyclosporin B [250]. Treatment of periodontitis and anti-microbial activity against pathogenic organisms to treat infectious diseases through SDT were successful, but still in pre-clinical stage. Further explorations may aid in identifying the optimal methodology for those conditions [357]. Additional management of atherosclerosis by SDT was also under rigorous exploration and scientists provide evidences for potential treatment of atherosclerotic plaques [358]. Certain drawbacks in SDT are the limitation with sound waves which possess properties such as scattering and diffraction. Unlike light waves in PDT, it remains difficult to restrict sound waves with in the region of interest. Inability of SDT to influence lungs which is an air bearing organ and the exposure time in SDT is usually longer, hence chance of adverse effects to become severe is possible. However, SDT is appreciated for its non-invasiveness and its selective targeting of cells compared to traditional treatments like chemotherapy or radiotherapy [356].

Site targeted drug delivery is one of the main challenges researchers faced during drug formulation. Constant exploration in this regard has led to several improvements in drug formulation with small drug delivery systems. Despite tremendous development in the drug delivery systems-based research, clinical safety was always a concern and hence drug delivery aided by an external force was sorted to avoid administration of any chemical-based drug delivery system. Use of ultrasound instrumentation is one among the successful methodologies that has shown significant success in site targeted delivery of therapeutic drugs developed for cancer, neurodegenerative diseases, endocrine disorders, infectious diseases etc [252,253].

Microbubble mediated drug delivery is one of the well-known and widely established method, which is constantly under research to make the therapeutic strategy better with further development to avoid the short comings and meet the challenges faced during treatment of different complex diseases [254]. Microbubbles can also be used as a contrast agent to obtain better signals and quality images; however, the nature of the bubbles was found to be suitable to load drug molecules with it for the targeted delivery with the ultrasound guided process [255]. MBs as contrast agents undergoes cavitation which results in size expansion or contraction, and the extent of this physical change influence the acoustic signals [359]. At low intensity, ultrasound cause vibration in the bubbles leading to minor changes in the vascular tissues and a permeability is created for the drug molecules to be delivered. The permeability of the blood vessels increases with increase in the ultrasound frequency [360]. Microbubbles are generally composed of a shell component covering a gas core. Some of the commonly used shell components of microbubbles includes lipids and derivatives or natural/synthetic polymers, while several contrast agents (gases) were tried and tested for encapsulation in the shell [361]. Use of microbubbles for drug delivery has gained significant research focus in the recent past and pre-clinical studies were conducted constantly in three different possible ways such as, drug loaded in to the microbubbles, in situ bubble formation from nanodrop as bottom-up approach and targeted microbubbles with ligand attached to surface [362].

Treatment of breast cancer was very well studied with respect to sonodynamic therapy. Some of them include, polymeric micelles made of Pluronic P123/F127 and loaded with curcumin to treat 4T1 breast cancer growth [363], DOX loaded PLA microbubbles, surface coated with TRAIL (Tumor necrosis factor-related apoptosis inducing ligand) has shown to inhibit the tumorigenic potential of MDA-MB-231 and MCF-7 ​cells [364]. Recently, gemcitabine encapsulated PLA bubble showed a potential against pancreatic cancer at the *in vitro* level, but failed to get translated *in vivo* and the researchers quoted that the concern was with drug loading capacity [365]. Quantum dots, nanoparticles like magnetic iron oxide and gold nanoparticles were also considered as a possible strategy for imaging and therapeutic modalities in ultrasound-based technologies [356,366]. Gene delivery through microbubble is one of the significant achievements in the field of US guided drug delivery systems. Mesoporous silica nanoparticles loaded with plasmid DNA encapsulated in a heterogenic co-polymeric shell was used to treat ovarian cancer in experimental mouse model, where the microbubble formulation not only reduced cancer growth but avoided non-specific cell damage [256]. O_2_ loaded microbubble coated with Span 60 and vitamin E was successful for oxygenation of breast cancer and making it radiosensitive for the radiation therapy [257].

Pancreatic ductal adenocarcinoma was successfully treated by sonosensitization and sonoporation mediated delivery of target drugs [282]. Logan et al. prepared lipid-based microbubbles coated with chemotherapeutic drug (gemcitabine, paclitaxel) encapsulated with perfluorobutane, showed 40% reduction in the tumor growth [258]. Similarly, DOX-loaded liposomes coated with magnetic iron oxide nanoparticles were encapsulated with perfluorooctane has resulted in enhanced delivery of the loaded drugs into the tumor site and reduced tumor growth up to 80% [367]. On the other hand, sonoporation was clinically approved for specific microbubble formulations (Optison, Definity, Lumason/SonoVue and Sonazoid) with optimal methodology (2.0 ​MHz of 20 μs pulses for 10 ​min at both high and low intensities). High power acoustic waves, weaken the endothelial junction and help in deeper penetration of the drugs. The Phase I clinical trial, resulted in tumor regression in 50% of the patients [259].

Sonoporation effect, leading to transient opening of BBB, plays a key role in success of drug delivery systems for Alzheimer's and Parkinson's diseases. Delivery of large molecules across BBB was for first demonstrated by Raymond and co-workers [260,261]. Further studies on sonoporation mediated BBB opening showed successful delivery of BAM-10 antibodies and improvement in behavioral pattern of the animals following Alzheimer's pathogenesis [193]. Similar studies were also reported on the successful delivery of RN2N tau specific antibodies [262], Ig delivery for downregulation of TNF-α and also MB mediated ultrasound affect the pathogenic processes like plaque reduction, improvement in cognitive and memory behaviour, etc [263]. The process of BBB transient opening was first patented by Lipsman et al. and Meng et al. specifically for the management of Alzheimer's disease [368,369]. Research studies pertaining to Parkinson's disease and ultrasound mediated sonoporation aided in successful gene therapy [193]. Viral vector mediated RNAi delivery by BBB opening showed significant improvement in the motor behaviour of the animals. Similarly, liposomal formulation, lipid-based microbubbles for delivery of genes that upregulate neurotrophic factors and showed potential treatment than nonconventional microbubble-based delivery [370,371]. Similarly, liposome mediated delivery of Nrf-2 showed significant neuroprotection [372]. Gasca-Salas et al., carried out a clinical trial with 5 patients administered with FDA approved microbubble formulation and MR guided FUS system [373]. Marked improvement in the cognitive function in the patients was observed without any adverse effects on the neurofunction due to BBB opening. Apart from the BBB opening, activation of astrocytes and microglia was also reported along with enhanced neurotransmitter delivery leading to neuromodulation. These changes also affect the homeostasis and helps in pro-inflammatory signals that could possibly help in β-amyloid clearance during Alzheimer's pathogenesis [193]. The neuro-suppressive activity of MB mediated ultrasound was reported due to increase delivery of GABA [374]. But, Cui et al. reported neurostimulation due to upregulated c-fos expression [375]. Clincal trial with MR guided FUS showed alterations in the neural circuits and neuromodulation [376]. Gene delivery-based increase in expression of Prestin has helped in better reception of ultrasound waves and improvement in neural activity [377]. Higher dose of MB and extended exposure to the ultrasonic waves has shown to upregulate the Nf-κB pathway, while 24 ​h post irradiation showed the expression of Nf-κB pathway related genes has returned to baseline [378,379]. Activation of innate immune cells like microglia and astrocytes in the neural system has also had an impact during ultrasound mediated BBB opening. This could pave way for exploring the possibility of immunomodulatory effects of US for treatment of cancer and other immune disorders that has influence on the nervous system [380]. Enhanced delivery of gentamicin was observed *in vitro* into bladder organoids after ultrasound irradiation and was effective against uropathogens, while better drug permeability into the intracellular compartment was observed when liposomes were directly sonicated than microbubble incorporation [381]. Cardiac graft rejection was reduced significantly when the anti-rejection drug was encapsulated into the liposome-based microbubble [382]. Ultrasound mediated microbubble disruption led to cavitation and increased susceptibility of urinary biofilm and catheter-associated biofilm to vancomycin [383]. Joint infection is one of the common challenges faced by the clinicians and pose a severe burden to the hospital community with yearly diagnosis of around 20,000 cases [384]. Researcher have found that, amikacin administration followed by ultrasound irradiation to burst microbubbles for enhanced delivery, has reduced the joint infections significantly and this was considered as a potential alternative for the septic arthritis treatment [385].

Identification of sonosensitizer property of certain compounds and their application potential in the SDT is a significant leap of success in the field disease management. However, they suffer with many limitations which includes poor pharmacokinetics, undesirable reaction in the skin and toxicity at high doses. Most of the sonosensitizers are hydrophobic in nature and they tend to get cleared of the system after its rapid aggregation in the physiological milieu. They are generally cleared by the immune system and a very meager number of drugs reach the desired target tissues. Due to inconsistent half-life of these molecules, it is difficult to ascertain the time of US application. Though the advancements in nanotechnology have helped these sensitizer compounds to overcoming these disadvantages, direct application of synthetic nanoparticles based sonosensitizers is not advisable and they should be chemically altered to suit the physiological environment without causing adverse reactions [386]. This is where the sensitizer molecules from biological origin are of great support and several indigenous molecules or formulations are explored in this regard. The most promising biomaterial identified were the exosomes, that mimic the nanoparticles with its physico-chemical characteristic and are primarily made of biomolecules.

#### 5.2.3 Ultrasound based theranostic applications

Ultrasound has become a very important technology that aids in diagnosis and therapy of many diseases that has complex intracellular pathways leading to pathogenesis. Treatment modalities that influence these pathways at the intracellular level can be very effective [387]. The research studies mentioned above showed that ultrasound can provide permeability to the drug molecules, apart from its excellent application in diagnosis. There are many such reports that provides evidence for this phenomenon and this makes ultrasound based theranostics one of the highly sorted technologies for treatment of complex diseases [388]. Microbubble with gas core influenced by acoustic waves plays the key role to render the diagnostic application and microbubble in this methodology is termed as ultrasound contrast agent [389]. Ultrasound responsive microbubbles decorated or encapsulated with variety of therapeutic formulations were developed and administered, so that ultrasonic waves when applied can help in enhanced release and entry of drug in to the region of interest, which was localized with the help of ultrasound imaging enhanced by the microbubbles filled with gases [203].

Molecular imaging with the ultrasound irradiation is of many types like Phase changeable imaging, photoacoustic imaging, and multimodal imaging, while recently tumor responsive imaging was discovered where gas production in response to ROS [390] and pH alteration at the target tumor site aiding in enhanced imaging [391]. Similarly, gas filled protein nanostructure with genetic information has the ability to enhance the quality of ultrasound images [343]. Phase changeable imaging works under the effect of cyclic acoustic waves application that changes the liquid phase of the contrast agents (perfluorocarbons) in to vapour phase and the process is termed as acoustic droplet vaporization. ADV has higher penetrating potential than optical droplet vaporization or NIR droplet vaporization [392]. Photoacoustic imaging involves the principle of thermal effect produced by photothermal transducing agent, which captures the light energy and converts it to thermal energy, which not only induced cancer cell death, but helps in accumulating and processing the data sets to obtain clinical information in the form of ultrasound images [393]. In case of multimodal imaging, silica nanoparticles with structural characteristics of microbubbles, is exploited as it possesses echogenicity and can also entrap the liquid or gas-based contrast agents that can further enhance the ultrasound signals. PFH based ultrasound contrast agents and magnetic nanoparticles with MR contrast properties can be encapsulated together in a mesoporous silica nanospheres which can be utilized for parallel MR, NIR and ultrasound imaging. Similar researches were conducted with variety of contrast agents for different imaging modalities to achieve multimodal imaging [394].

As we discussed earlier about several number of evidences for ultrasound-based therapy, and development in technologies for enhancing the ultrasound images using contrast agent, the focus on several theranostic applications of ultrasound is inevitable. Nanotechnology has greatly contributed to development of such theranotic platform [395]. Combination of mechanical and thermal effects were considered as the key component for enhancing the drug delivery and drug sensitization [396]. Mechanical effects exerted by ultrasound includes cavitation, acoustic radiation force and acoustic streaming [397]. The radiation force generated by ultrasound was called Bjerknes force which has the ability to reduce the speed of the flowing contrast agent, by moving the microbubbles that are generated by the acoustic radiation force, to the wall of the blood vessels [398,399]. The intravascular transducer was used to displace the microbubble to the vessel wall and aggregate to help in drug delivery along with obtaining enhanced ultrasound imaging [400]. There are number of reflecting and scattering materials/objects, occur in the field of ultrasound and bulk streaming is a phenomenon of aligning those objects in the direction of the propagating ultrasonic radiation forces. While circulating movement of the fluid around the cavitating particles are called microstreaming effect. These mechanical streaming effect leads to altered velocity of blood flow and movement of particles in the blood flow, which will greatly help in enhancing the acoustic signal and delivery of drug to the target site [396]. On the other hand, generation of thermal energy is also a consequence of ultrasound irradiation which pass through tissue and generate friction [207]. Hyperthermia has been effective in ablation of tumor mass and certainly increased the therapeutic efficacy of the chemo drugs. Appreciable amount of hyperthermia generated by acoustic waves is considered useful for drug cargo to the target site and sensitization to improve efficacy [396,401].

Biological and nanomedicine-based application of ultrasound has been established several decades before and is under constant scrutiny to avoid the challenges and improve the output of imaging. Common challenges like poor tissue penetration, interference from neighboring tissues during ultrasound received back at the transducers. Several complexities in diagnostic process after imaging was also constantly addressed and resulted in many combinatorial imaging strategies. On the other hand, notable number of studies were reported on the therapeutic potential of ultrasound and acoustic systems. Similar to imaging methods, therapeutic methods were also approached critically to reduce the side effects like nonspecific cellular damage. Hence, US based imaging and guided therapy has become one of the routinely sorted theranostic strategies. Development of suitable ultrasound contrast agents is being constantly explored and on the other hand, potentiating the exosomal theranostic property has become absolutely needed as the challenges faced during the *in vivo* applications, weigh down the excellent biological property of exosomes.

## 6 Ultrasound enhances the theranostic property of exosomes

Application of exosomes in diagnosis, drug delivery and enhanced therapeutic efficacy has shown a lot of advantages over synthetic formulations like liposomal and other nanocarriers. Exosomes consists of naturally occurring micro/macromolecules that can be exploited as natural contrast agent or drug delivery vehicles [122]. Biochemical profile of exosome has the ability to influence major physiological events during disease progression hence, this gives an inherent ability to the exosomes as a drug delivery system [402], while biochemical and physicochemical characteristics of exosomal surface aid in tracking them during systemic circulation and tissue localization [176]. Utilization of exosomes as theranostic agent has gained interest recently, as it can aid in personalized and clinically optimal medication for complex diseases like cancer, neurodegenerative diseases, and endocrine disorders [403]. Exosomes with its excellent properties can also help in guided therapy and further their submicron size makes it much more relevant and feasible for enhanced drug delivery to the targeted site [404]. On the other hand, it is a fact that any externally applied physical or chemical force can potentiate the efficacy of the drug formulation. Hence, several therapeutic strategies were influenced by certain physical energy like light, sound, or heat. In this regard exosomes can also be chemically influenced by engineering their core and surface in order to facilitate targeting and delivery [405]. Among the well-established and much accepted external influencing force, ultrasound has shown significant success and the pertaining methodologies were constantly developed and updated for improving the therapeutic potential [126]. In this section we would like to discuss how ultrasound influences exosomal physical and chemical properties so that its theranostic potential has been improved much better than without the acoustic stimulus.

### 6.1 Isolation of exosomes

**Fig. 3:**
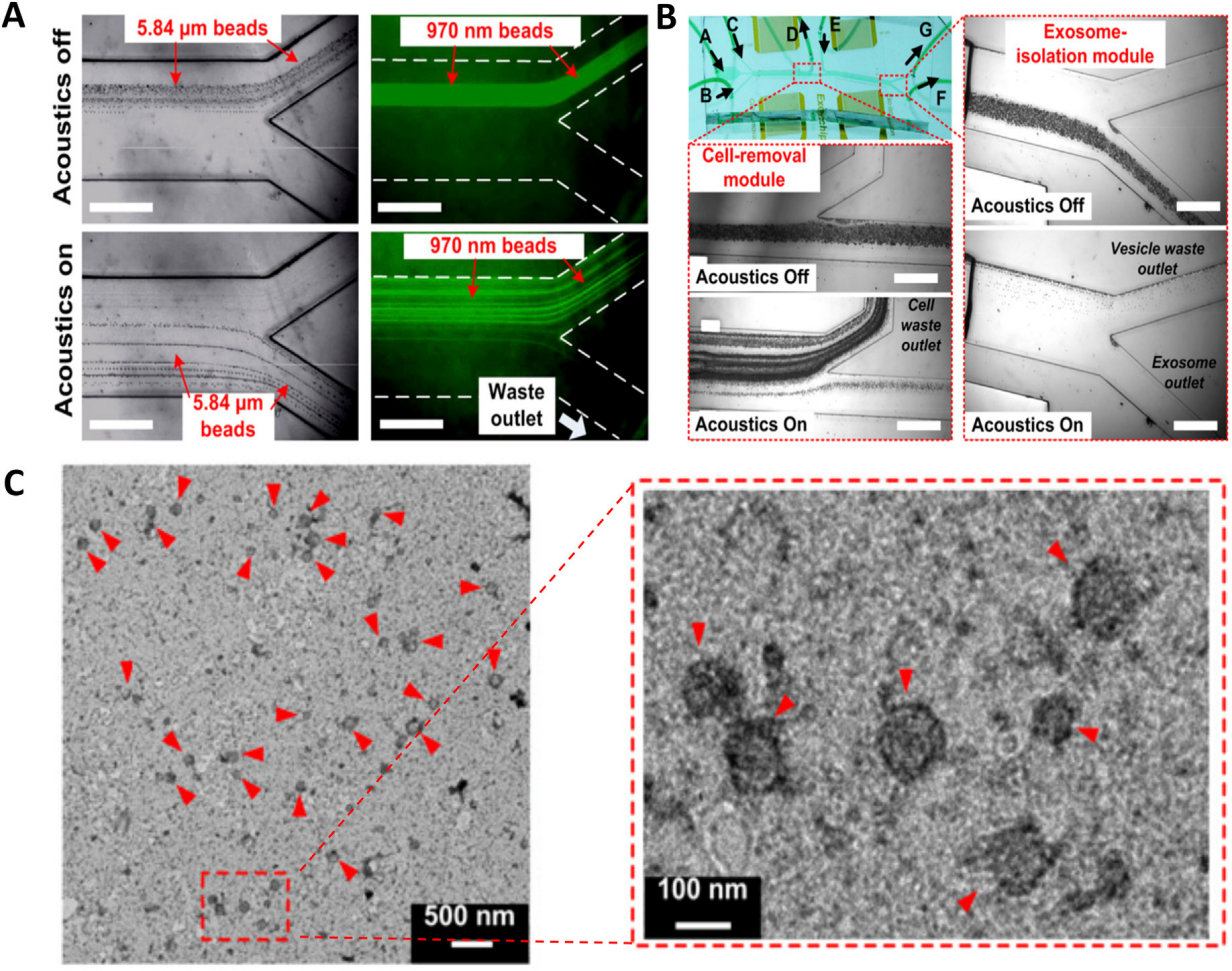
**Illustrations on the effect of acoustic waves is separation of exosomes from other blood components.** (A) Separation of synthetic microparticles and submicrometer particles using the acoustofluidic cell-removal module. Polystyrene particles with diameters of 5.84 ​μm (not labeled) and 970 ​nm (labeled with Dragon Green fluorescent dye) were processed through the acoustic field. The taSSAW field deflected microparticles to the waste outlets. The acoustic radiation force was not sufficiently large to move the submicrometer particles, which were therefore separated from microparticles at the outlet. White stripe in the two left panels indicates the centerline location of the CCD (charge-coupled device) image sensor. (Scale bar: 500 ​μm). (B) Isolation of exosomes from whole blood using the integrated device using acoustofluidics. In the experiments, inlet A is for whole blood; inlets B, C, and E are for sheath flows. Outlet D is cell waste. Outlets F and G are for isolated exosomes and vesicle waste, respectively. Images were taken under the microscope at the corresponding areas of the device. Blood components were directed to each corresponding outlet when the acoustic wave was on. White stripe in the four grayscale panels indicates the centerline location of the CCD image sensor. (Scale bar: 500 ​μm). (C)TEM images of isolated exosomes. The exosomes (red arrows) have a characteristic round shape and a cup-like structure. The image was reproduced from the article published in The Proceedings of the National Academy of Sciences (PNAS) by Mengxi Wu, Tony J Huang and Co-workers in the year 2017 [412]. Permission was obtained from the publisher. (For interpretation of the references to colour in this figure legend, the reader is referred to the Web version of this article.)

Microfluidics based exosome isolation is one of the advanced techniques which is certainly successful. There has been constant upgradation in the methodology and combining certain external force to microfluidics has improved the efficiency and yield of the exosomes to many folds. Incorporation of ultrasound instrumentation, with piezo-acutators has become one of the successfully developed methods recently [185]. Acoustic waves employed in isolation of exosomes were of two types: bulk acoustic waves that generates mechanical vibrations and has ability to travel only between the transducer and the reflecting surface. However, the surface acoustic waves were produced by interdigital transducer and is more advantageous during addition with microfluidic technology [406,407]. Continuous acoustic streaming or trapping the exosomes using acoutic waves have been the currently identified and found to be successful during isolation of exosomes [408]. During the acoustic trapping process, stationary ultrasound irradiation creating the acoustic potential at a single point in the fluidic stream causes consecutive trapping of the exosomes and other components from the seed particles [409]. Theory behind the isolation process is that, particles bigger than the cutoff size that acoustic signals can trap will be retained and smaller particles will be gathered in the outlet. Several advantages were reported with respect to acoustic trapping like reduced time period and more importantly the biophysical and biochemical characteristics of the exosomes were preserved, which can aid in the diagnostic biomarker applications of exosomes [185,410]. Acoustofluidics was applied in conjunction with the stream of the fluidic channel that separates particles with different physical properties with the help of varied pressure fields. Hence, based on the physical characteristics like size, density, etc., the exosomes were separated from the other particles [408]. Recently the combination of piezoelectric effect and thermal effects was applied to isolate the exosomes with thermo-acoustophoresis approach [411]. Further Wu et al. has developed a fluidic technology where two sequential unit of ultrasonic transducers were constructed to first remove the cellular components followed by separation of exosomes in the second compartment and the crucial advantage of this process is the liberty to use undiluted human blood [412] (Fig. 3).

### 6.2 Exosomes for contrast enhanced ultrasound imaging

Responsiveness of exosomes to acoustic signals has created the interest in several researchers and led to series of development in ultrasound image enhancing contrast agent in the past few decades [413]. Exosomes were believed to fill the gaps between the disease progression and early & specific diagnosis [403]. Similar to drug delivery approaches, the exosomal loading of contrast agents or labelling dye to obtain improved and targeted imaging. Osborn and co-workers have developed exosomes to responsed to ultrasonic signals. They measured the linear and non-linear scatter of the response signals obtained from echogenic exosomes and applied to image them in an *in vivo* setup. The results showed significant increase in brightness with echogenic exosomes while, the preparation method of the echogenic exosomes, involved series of freeze-thawing and freeze drying cycle in the presence of mannitol [414]. Unlike, the echogenic liposomes encapsulated with bigger sized microbubbles, the echogenic exosomes were preformed vesicles, and are in submicron size [415]. The small nanocups resembling exosomes, upon acoustic excitation, changes its surface characteristics and undergo size growth with surface trapped bubble. The key step in generation and application of echogenic exosomes, is freeze drying with mannitol and reconstituting them in a suitable media as it influences the echogenic behaviour [416].

### 6.3 Bioengineering of exosomes

Biological origin of exosomes makes it more stable in the dynamic *in vivo* system and feasible to evade the toxic response provided by the incorporated drugs. The diagnostic, targeted delivery and therapeutic potential of exosomes was mostly exploited in the theranostic platform exclusively or after engineering with desired drug molecules [157,417]. The engineering of exosomes can be performed by many methods like, passive loading, modification with certain physical methods, internalization of certain nucleic acids by chemical modification, surface labelling with target molecules and photoassisted loading [96,418,419]. Among the physical methods explored, sonciation was a much anticipated and successful strategy, but is still underexplored with respect to development of an optimized protocol to obtain structurally unaltered exosomes. With enormous amount of reports referring to success of exosomes in cancer therapeutics, most of the sonication-assisted bioengineering of exosomes were performed with chemotherapeutic drugs [418,420].

**Fig. 4:**
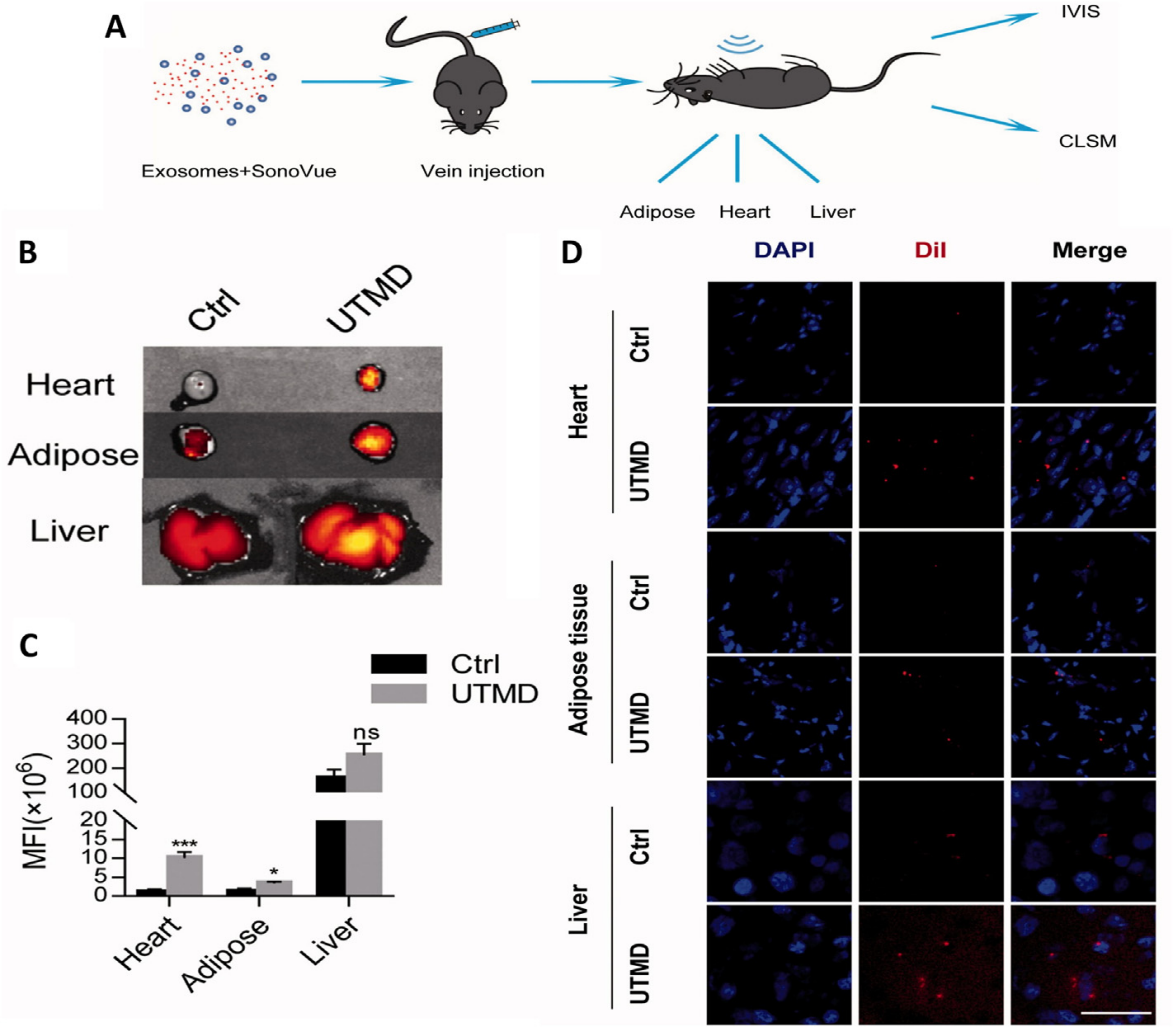
**UTMD promotes the uptake of injected exosomes in the targeted tissues.** (A) Schematic representation of the experimental procedure. Labeled exosomes, together with the SonoVueTM microbubbles were injected via tail vein. Distribution of the labeled exosomes was tracked by fluorescence imaging. (B) Fluorescence signal intensity in the tissues (in heart, adipose tissue and liver) with or without UTMD. (C) Quantification of Fluorescent signal. (D) CSML image revealing the increased uptake of DiI-labeled exosomes in indicated tissues by UTMD. Nuclei were counterstained with Hoechst and scale bar ​= ​50 ​μm. The image was reproduced from the article published in Drug Delivery Journal by Sun, Li, Yuan and Co-workers in the year 2019 [425]. Permission was obtained from the publisher for using the illustration.

**Fig. 5:**
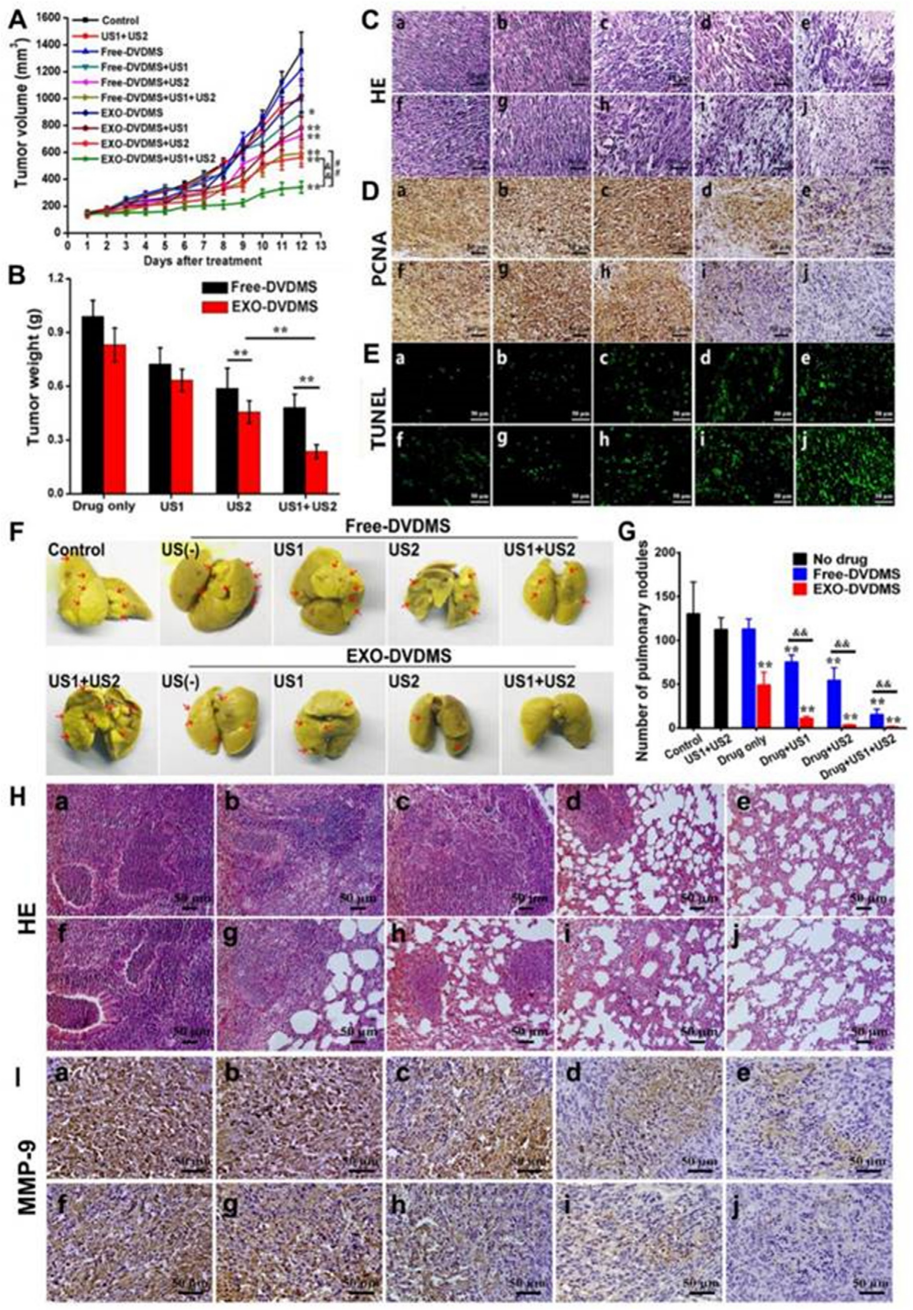
**In vivo enhanced therapeutic effect and lung metastasis suppression of EXO-DVDMS-SDT in 4T1-xenograft mice.** (A) Tumor growth curves of different groups of 4T1 tumor-bearing mice (n ​= ​6). ∗∗p ​< ​0.01 versus control, ##p ​< ​0.01 versus Free-DVDMS ​+ ​US1+US2, && p ​< ​0.01 versus EXO-DVDMS ​+ ​US2. (B) Tumor weight inhibition ratio at the 12th day of different treatment groups. ∗∗p ​< ​0.01 between different groups. (C) H&E stained images of tumor sections collected from different treated groups of mice. Bar ​= ​50 ​μm. (D) Immunohistochemistry detection for PCNA after different treatments. Bar ​= ​50 ​μm. (E) In situ apoptosis by TUNEL assay. Bar ​= ​50 ​μm. (F) Photos of lungs after soaking in Bouin's solution showing spontaneous pulmonary breast cancer metastases (red arrows). The mouse lungs were taken at the 12th post different treatments and the gross appearance of pulmonary nodules was photographed. (G) The pulmonary nodules were manually counted and the average numbers were calculated in different groups. ∗∗p ​< ​0.01 versus control, && p ​< ​0.01 EXO-DVDMS versus Free-DVDMS. Data shown are mean ​± ​S.D. of five batches. (H) H&E stained images of lung sections collected from different treated groups of mice. Bar ​= ​50 ​μm. (I) Immunohistochemistry detection for MMP-9 level of 4T1 tumors after different treatments. (a) Control, (b) US1 (2 ​W, 3 ​min)+US2 (3 ​W, 3 ​min), (c) Free-DVDMS (2 ​mg/kg), (d) EXO-DVDMS (2 ​mg/kg), (e) Free-DVDMS ​+ ​US1, (f) EXO-DVDMS ​+ ​US1, (g) Free-DVDMS ​+ ​US2, (h) EXO-DVDMS ​+ ​US2, (i) Free-DVDMS ​+ ​US1+US2, (j) EXO-DVDMS ​+ ​US1+US2. The image was reproduced from the article published in Theranostics Journal by Liu, Bai, Wang and Co-workers in the year 2019 [426]. Permission was obtained from the publisher for using the illustration. (For interpretation of the references to colour in this figure legend, the reader is referred to the Web version of this article.)

Doxorubicin and paclitaxel were successfully loaded drugs in the exosomes, where the sonication period followed by an hour of incubation helped in restructuring of the exosomal membrane. This study showed ultrasound assisted drug loading was more effective than the other methods like electroporation and passive loading [114,421,422]. Similarly, treatment of pancreatic cancer with exosomes loaded with gemcitabine was successful and much better compared to passive loading, when the sonication was incorporated. However, the method was not highly sorted because, the physical damage caused to the exosomal membrane was more compared to other methods and it concerns the researchers to develop a optimal protocol to avoid damage to the membrane [418,423,424]. Acoustic waves were also used for the exosomes-based drug delivery modalities which involves gaseous microbubble, that was either structurally manipulated with low pressure ultrasound (stable cavitation) or it was burst and collapsed with high pressure ultrasound (inertial cavitation). Fluorescent dye (DiR/Dil) labeled exosomes, when injected into mice with and without the combination of microbubble revealed that, ultrasound targeted destruction of microbubble (UTMD) at the desired region aided in internalization of the exosomes by endocytosis (Fig. 4) and this was feasible only for an optimal time duration of ultrasound irradiation [425]. Exosomes engineered with sonosensitizer (sinoporphyrin sodium) was successful in targetted delivery of exosomes and further deep tissue penetration and sustained release was also achieved by focussed ultrasound (Fig. 5) [426].

### 6.4 Exosomes mediated drug delivery

Exosomal drug delivery as discussed earlier has showed significant potential and success recently, but hasn't reached clinical translation stage due to several disadvantages [23]. Some of major concerns raised by experts are the efficiency of targeting and release of drug components [427]. Researchers has facilitated the exosomal drug sensitization by application of external energy like light, heat and sound. Though, each methodology involving external force has their own share of advantages and disadvantages, the ability of drug targeting and release was significantly higher [419].

**Fig. 6:**
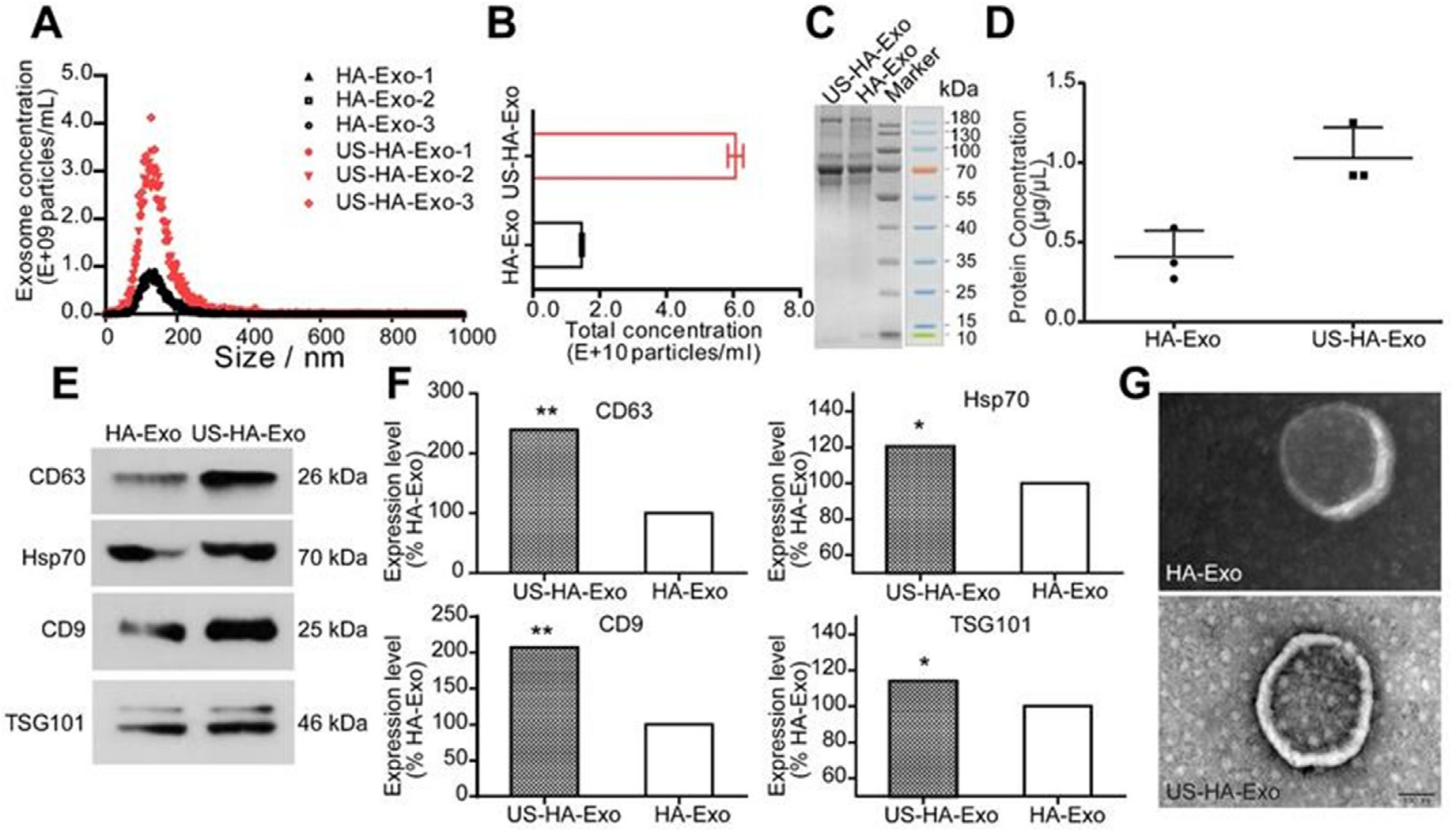
**Characterization and quantification of astrocyte-derived exosomes.** (A) Nanoparticle tracking analysis of ultrasound-stimulated astrocytes and astrocyte-derived exosomes showing the number and size distribution and (B) Total concentrations. Total protein level of exosomes determined by SDS-PAGE (C) and BCA (D). Western blotting (E) and densitometric quantification (F) of biomarker proteins of exosomes from both ultrasound-stimulated astrocytes and astrocytes without any treatment. (G) Representative TEM of exosomes. Scale bars: 100 ​nm ∗ denotes significant difference compared with controls. Data are presented as means ​± ​SD (n ​= ​3). ∗P ​< ​0.05; ∗∗P ​< ​0.01. The image was reproduced from the article published in Theranostics Journal by Deng, Wang and Meng and Co-workers in the year 2021 [428]. Permission was obtained from the publisher for using the illustration.

**Fig. 7:**
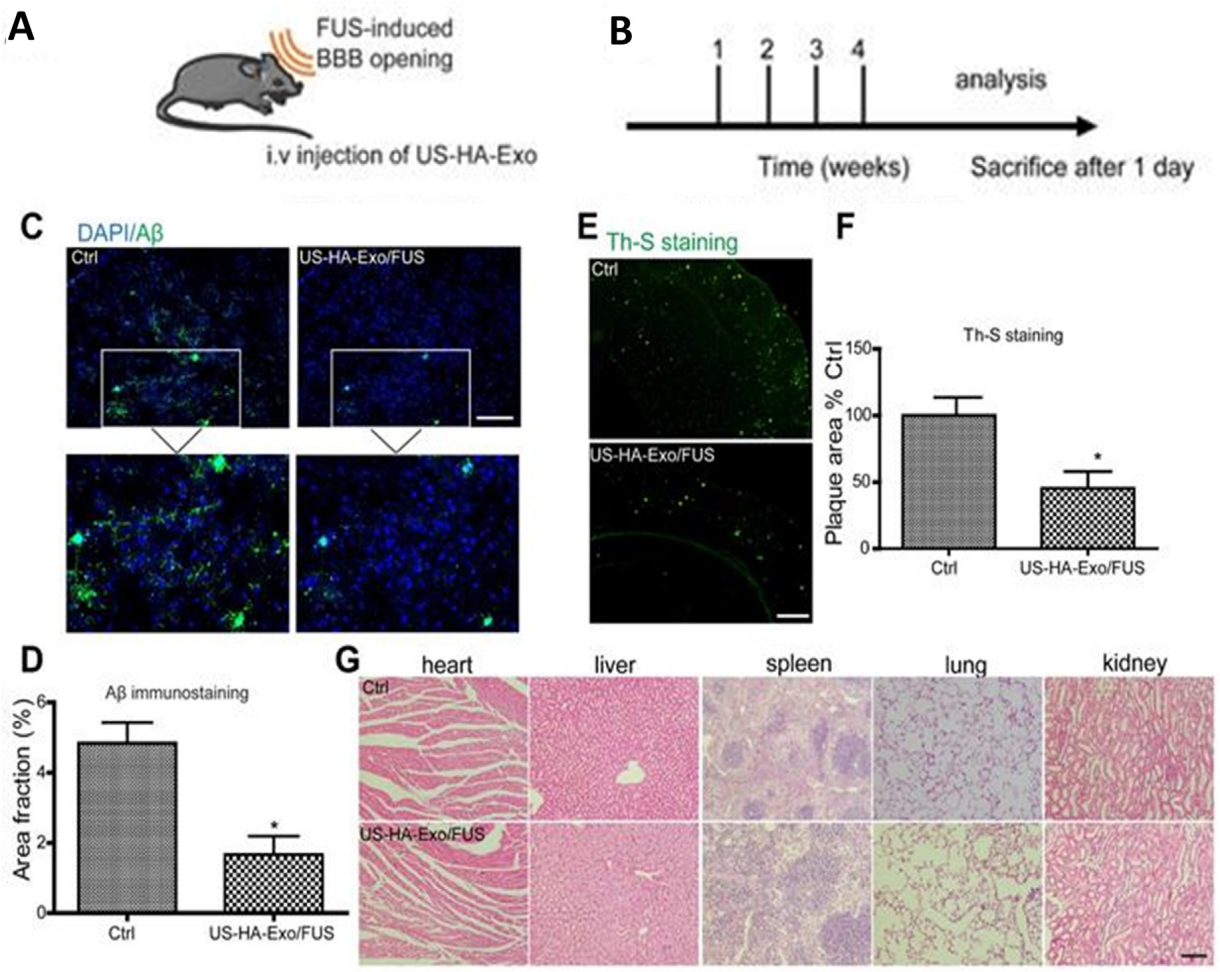
**Brain-targeted delivery of US-HA-Exo combined with FUS-mediated BBB opening.** (A, B) Schematic of FUS-BBB opening-assisted delivery of exosomes. (C) Brain sections obtained from 10-month-old APP/PS1 mice were immuno-stained for Aβ, scale bar: 100 ​μm. (D) The percentage area of positive amyloid-β staining was quantified. (E, F) Aβ plaques in the brain were detected by thioflavin-S staining (E; scale bar: 100 ​μm), and quantified (F). (G) H&E staining of major organs of mice, scale bar: 100 ​μm. The image was reproduced from the article published in Theranostics Journal by Deng, Wang and Meng and Co-workers in the year 2021 [428]. Permission was obtained from the publisher for using the illustration.

Ultrasound mediated exosomal diagnosis/therapy has aided in treatment methods for several diseases [405]. Ultrasound mediated brain delivery of exosomes is well established as the acoustic waves aids in transient opening of BBB. Deng et al. in the year 2021 has delivered the exosomes from astrocytes and administered in mice for improvement of Aβ induced neuro toxicity. This study gives a valid support in favour of ultrasound and its help in isolation of exosomes from astrocytes (Fig. 6) and further it discusses about the ability of ultrasound in exosomal delivery across the BBB (Fig. 7) [428].

**Fig. 8:**
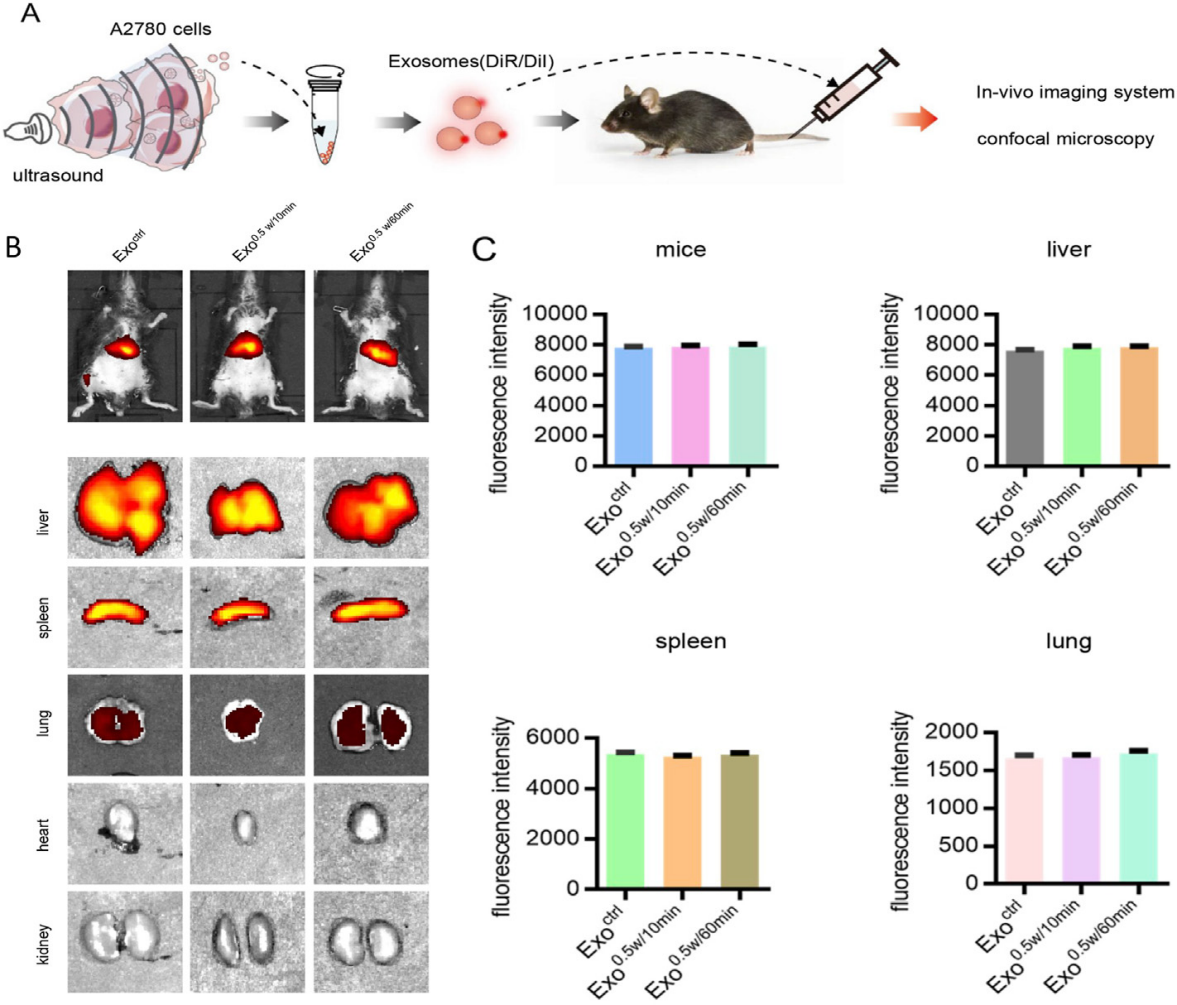
**Effect of ultrasound on distribution/accumulation of exosomes labeled with DiR.** LIUS induced exosomes have similar *in vivo* distribution profile. (A) Schematic diagram of the experimental procedure. (B) Exosomes from cells treated with or without LIUS were labeled with DiR. About 100 ​μg (at protein level) of exosomes in 100 ​μL were injected to C57BL/6J mice via the tail vein. Ex vivo imaging was analyzed by IVIS and data were representative images of the data from three mice. (C) Statistical analysis of fluorescence intensity in whole mice, liver, spleen and lung. n ​= ​3. No significant differences were found. The image was reproduced from the article published in Journal of Drug Delivery Science and Technology by Zhao, Chen, Hao and Co-workers in the year 2020 [431]. Permission was obtained from the publisher for using the illustration.

**Fig. 9:**
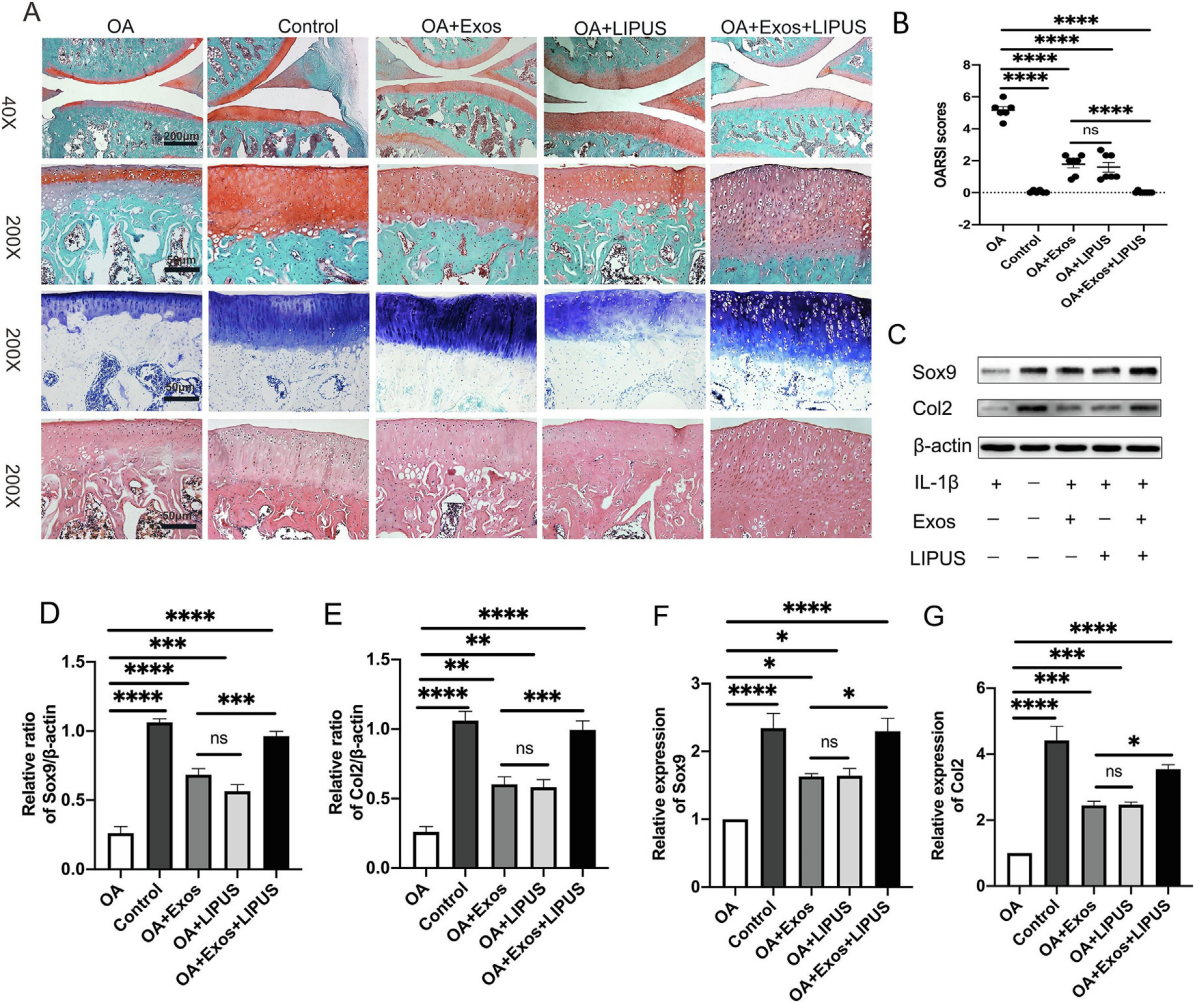
**LIPUS strengthens the promotion of BMSC-derived exosomes on osteoarthritic cartilage regeneration.** (A) Safranin O/Fast Green, Toluidine Blue and H&E-stained sections of knee joints. Scale bar: 200 ​μm (40 ​× ​), 50 ​μm (200 ​× ​). (B) Cartilage degeneration evaluated with the OARSI scoring system. (n ​= ​6, 6, 7, 7, 8 respectively for control, OA, OA ​+ ​Exos, OA ​+ ​LIPUS, OA ​+ ​Exos ​+ ​LIPUS group). ns P ​> ​0.05, ∗∗∗∗P ​< ​0.0001. (C) Chondrogenesis-related protein level were detected by western blot. Protein (D and E) and mRNA (F and G) expression of Sox9 and Col2 were measured by western blot and qPCR, and the results of statistical analysis of three independent experiments are revealed. ns P ​> ​0.05, ∗P ​< ​0.05, ∗∗P ​< ​0.01, ∗∗∗P ​< ​0.001, ∗∗∗∗P ​< ​0.0001. The image was reproduced from the article published in International Immunopharmacology Journal by Liao, Li, Yuan and Co-workers in the year 2021 [433]. Permission was obtained from the publisher for using the illustration. (For interpretation of the references to colour in this figure legend, the reader is referred to the Web version of this article.)

Fluorescent dye and doxorubicin incorporated exosomes and their penetration across the blood brain barrier was facilitated by focussed ultrasound (FUS) application for treatment of orthotopic glioma in mice [429]. Low intensity pulsed ultrasound (LIPUS) methodology was applied on bone marrow derived dendritic cells and the exosomes isolated from LIPUS treated and untreated cells. The isolated dendritic cells’ exosomes were labeled with human umbilical vein endothelial cells (HUVECs). Upon addition of TNF-α, the reduced expression of intercellular adhesion molecule-1 (ICAM-1) and vascular cell adhesion molecules-1 (VCAM-1) was observed in the LIPUS treated dendritic cell exosomes compared to untreated samples where it was significantly upregulated. On the other hand, increased expression of miRNA-16 and 21 in exosomes obtained from ultrasound treated dendritic cells, resulted in significant reduction in NF-κB signaling pathway and downregulation of TNF-α mediated endothelial inflammation [405,430]. Apart from this, release of exosomes was also aided by low intensity ultrasound in ovarian cancer cells (Fig. 8), where 60 ​min exposure of acoustic waves led to increased exosome release without any change in structural, molecular and biological properties [431]. Lung cancer was also successfully treated by exosome targetted to specific tissue with the help of ultrasound irradiation. Apart from this ability of BMSC derived exosomes to alleviate the osteoarthritis and bone regeneration was further potentiated by low intensity US (Fig. 9) [432]. Scientists believed that mechanism of ultrasound triggered exosomes release by cells could be through influencing the molecular machinery (ESCRT complex, Rab GTPases and TSAP genes) involved in exosomes biogenesis pathway. The strategy of ultrasound assisted exosome release can be successfully applied in a bioreactor for large scale production of exosomes [433].

## 7 Conclusion and future perspectives

Exosomes are excellent biological entity that has the ability to influence vital biological processes which can alter the physiological and pathological processes in the human systems. Scientists have utilized the functions of exosomes for various biological and clinical applications like diagnosis and therapy of complex diseases. However, efficiency of the applications is not up to the mark as expected because of the poor specificity of the naïve exosomes and loss of the biological property during the engineering of exosomes. Hence, it is essential to improve the efficacy of both naïve and engineered exosomes and this can be achieved by application of certain external energy that can improve biological stability and property. Ultrasound is one of the well-established imaging modalities and its principle was not only used for image-based diagnosis but was also utilized to improve therapeutic processes. The therapeutic applications involving ultrasound are collectively called as sonodynamic therapy and it includes targeted drug delivery, drug sensitization, release and internalization that are successfully improved by ultrasound waves. Important therapeutic methods includes nanoparticle-based drug formulation, some of which are in clinical trial phase and excellent responsiveness of the nanoformulation to acoustic waves was documented in those studies. This prompted experts to apply ultrasound-based strategy for improvement of exosome-based therapy. Exosome production and targeted delivery of the therapeutic exosomes were assisted by the ultrasound had a significant effect against Aβ toxicity and Alzheimer's progression, while cancer management, bone tissue regeneration and etc were improved by therapeutic exosomes. Further, exosomes encapsulated in microbubbles were used for contrast enhanced *in vivo* tracking of exosomes and aid in targetted therapy [425,426,428,432].

Though the hypothesis has been tested with exosomes from several cell types and for various disease management, the ultrasound assisted exosomal therapy has not been translated yet to the clinical set up. This may be due to several reasons such as the poor scalability of the exosome isolation for clinical studies, inability to retain the structural and molecular integrity of exosomes under ultrasound irradiation, issues with target specificity, inability of sonosensitization protocols for deep tissues due to limited penetration of the ultrasound and systemic stability of exosomes after administration. Hence, continuous research explorations are carried out to address the issues as the *in vitro* and *in vivo* reports are in favour the methodology. Currently explorations are conducted to fill the gaps like optimizing the route of administration, preparation of formulation with excellent *in vivo* stability, improving the target specificity, engineering the exosomes with out altering its natural biophysical and biochemical characteristics and etc. Crossing the immune check points is one of the important stage of any drug formulation and experts are focusing in this area for improving the stability of exosomes to evade the immune response mediated destruction of exosomal therapeutics. Bioengineering of exosomes is one of the much discussed aspects of their therapeutic applications and very less has been discussed about the surface modification and internalization of exosomes with effector molecules to enable the diagnostic imaging such as CT, MRI apart from US. Great interest has been developed regarding diagnostic applications of engineered exosomes with imaging contrast agents. At the same time the protocols of the imaging modality including US should be adjusted in such a way that it doesn't affect the properties of the exosomes and also the acoustic waves mediated cell damage can also be avoided. Future research should be focussed towards establishing ultrasound assisted exosomes as the most common theranostic platform which is safer and effective in disease managements.

## Author contributions

**Badrinathan Sridharan:** Conceptualization, Data Curation, Writing - Original Draft, Writing - Review & Editing. **Hae Gyun Lim:** Conceptualization, Resources, Writing - Review & Editing, Supervision, Project administration, Funding acquisition.

## Funding information

This work is supported in part by Samsung Research Funding & Incubation Center of Samsung Electronics under project number SRFC-IT2202-02 and in part by the National Research Foundation of Korea (NRF) grant funded by the Ministry of Science and ICT under project number 2022R1A5A8023404.

## Declaration of competing interest

The authors declare that they have no known competing financial interests or personal relationships that could have appeared to influence the work reported in this paper.

## Data Availability

No data was used for the research described in the article.
